# Environmental drivers of metapopulation dynamics throughout the full annual cycle in a declining Arctic‐nesting migratory herbivore

**DOI:** 10.1111/1365-2656.70236

**Published:** 2026-03-05

**Authors:** Alexander R. Schindler, Anthony D. Fox, Alyn J. Walsh, Larry R. Griffin, Seán B. A. Kelly, Mitch D. Weegman

**Affiliations:** ^1^ Department of Biology University of Saskatchewan Saskatoon Saskatchewan Canada; ^2^ Department of Ecoscience Aarhus University Aarhus Denmark; ^3^ National Parks and Wildlife Service Dublin Ireland; ^4^ Wildfowl & Wetlands Trust Gloucester UK; ^5^ ECO‐LG Limited Dumfries UK

**Keywords:** capture–recapture, climate change, demography, dispersal, integrated population model, subpopulations

## Abstract

1. Assessing the impacts of changing environmental conditions on animal species requires thorough understanding of population dynamics, which can be difficult to estimate when animals aggregate into spatially discrete subpopulations.
2. We used 39 years of fecundity, capture–recapture and abundance data in an integrated metapopulation model to study environmental drivers of demography in a declining migratory bird, the Greenland white‐fronted goose (*Anser albifrons flavirostris*).
3. We found that low fecundity due to earlier spring vegetation phenology on staging areas and increased snow on breeding areas explained metapopulation decline, though the strength of these effects varied by subpopulation. Differential immigration and emigration rates affected local wintering abundance trends, highlighting the importance of quantifying subpopulation‐metapopulation dynamics for understanding fragmented animal populations.
4. We provide a framework for extending commonly used integrated population models to a metapopulation framework for testing novel ecological hypotheses about how changing environmental conditions within and among subpopulations drive changes in animal abundance.

## Graphical abstract

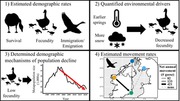

## 1 INTRODUCTION

Quantifying demographic properties (e.g. survival, fecundity and movement) and their environmental drivers is crucial to understanding how animal populations change over time (Frederiksen et al., 2014; Letcher et al., 2015; Warlick et al., 2024). However, demographic rates often vary within animal populations, complicating our understanding of population dynamics. Varying environmental conditions within a metapopulation may create heterogeneity in survival, fecundity, emigration and immigration rates among subpopulations (Hanski, 1998; Hanski & Gilpin, 1991; Levins, 1969). Source (i.e. growing) subpopulations, where births and/or immigrants exceed deaths and/or emigrants, may provide individuals that disperse to sinks (i.e. subpopulations where deaths and/or emigrants exceed births and/or immigrants, which decline to extinction without immigration; Boughton, 1999; Pulliam, 1988). Quantifying subpopulation demographics and source‐sink dynamics helps us understand how survival, fecundity and movement among subpopulations differentially contribute to overall metapopulation growth rate (Pulliam & Danielson, 1991), aiding conservation prioritisation that maximises population viability. However, detailed assessments of subpopulation demography and source‐sink status remain scarce (Furrer & Pasinelli, 2016), resulting in conservation planning based on local demographic information, without estimation of heterogeneity in survival, fecundity and dispersal rates among subpopulations (Millon et al., 2019).

Metapopulation theory has traditionally been applied to primarily studying population dynamics of nonmigratory animal species (Esler, 2000). When studies do apply metapopulation concepts to migratory systems, many consider subpopulations as spatially disjunct breeding units, which may mix during other times of the year (e.g. Alisauskas et al., 2022; Rushing et al., 2016). However, a subpopulation‐metapopulation framework can similarly be applied to other phases of the annual cycle when spatial, temporal or behavioural mechanisms create demographically independent subpopulations (Esler, 2000; Taylor & Hall, 2012). For example, species with high migratory connectivity are characterised by a large proportion of individuals from the same breeding areas migrating to the same wintering areas along similar routes with limited mixing of individuals from other regions (Cohen et al., 2018). In such cases, subpopulations of migratory animals maintain demographic independency throughout the annual cycle and metapopulation structure is preserved during nonbreeding periods. Although population dynamics of migratory birds are traditionally studied using breeding season data (Hostetler et al., 2015), nonbreeding (i.e. winter) studies may be more practical if breeding areas are inaccessible to researchers or nonbreeding areas are emphasised in conservation planning.

Integrated population models (IPMs) quantify animal population dynamics by combining multiple types of population‐level data, including count, capture–recapture and fecundity information (Schaub & Abadi, 2011) to generate more accurate and precise estimates of population size, survival, fecundity and movement rates than simpler models, even when demographic data are incomplete (Kéry & Schaub, 2012). Most published IPMs have focussed on a single population, either a ‘local’ population where demographic rates are reasonably assumed to be homogeneous across space or a ‘global’ population, which allows inference about average demographic rates across the species' range (Schaub & Kéry, 2021). With the growing availability of spatially replicated population monitoring data, researchers are increasingly accounting for spatial variation in the demographic parameters governing population dynamics when developing IPMs, often by representing space in a continuous (Chandler et al., 2018; Chandler & Clark, 2014; Sun et al., 2019) or near‐continuous way (e.g. by dividing space into smaller spatial units or strata; Ahrestani et al., 2017; Plard et al., 2020; Zhao, 2020). When animals are patchily distributed, discrete parameterisations of space are useful for studying population dynamics. Integrated metapopulation models (IMMs) use a metapopulation framework for parameterising IPMs and account for demographic heterogeneity among discrete subpopulations with dispersal among them (McCrea et al., 2010; Péron et al., 2010; Seward et al., 2019). With anthropogenic land‐use change increasing habitat fragmentation for many animal species, IMMs are a valuable tool for understanding how and why animal populations change across space and time. However, IMMs remain rare, particularly for migratory species with broad seasonal ranges.

We developed an IMM to quantify spatiotemporal patterns in demography of a long‐distance migratory bird of conservation concern, the Greenland white‐fronted goose (*Anser albifrons flavirostris*). Greenland white‐fronted geese breed in west Greenland, stage in south and west Iceland during spring and autumn migration, and in winter aggregate at 68 discrete wintering sites (i.e. subpopulations) in Great Britain and Ireland, likely due to uneven food distribution (Fox et al., 1999; Malecki et al., 2000; Ruttledge & Ogilvie, 1979). Geese show high winter site fidelity, with most adult individuals returning to the wintering sites where they were marked (Warren et al., 1992; Weegman et al., 2015; Wilson et al., 1991), although some dispersal occurs; 14%–15% of marked individuals were observed changing wintering sites each year and 22% observed changing wintering sites at least once during their lifetime (Warren et al., 1992; Weegman et al., 2015; Wilson et al., 1991). Greenland white‐fronted geese also demonstrate high migratory connectivity, and thus, wintering distribution is indicative of population structure during staging and breeding phases as well (Fox et al., 1983; Francis & Fox, 1987; Kampp et al., 1988). Because these geese breed at low densities in remote Arctic areas, most research and conservation efforts over the last 50+ years have occurred on the wintering areas. These features suggest that the demography of Greenland white‐fronted geese should be estimated in a winter‐based metapopulation framework whereby subpopulation dynamics are discretely estimated and connected among subpopulations. From 1999 to 2022, the total number of Greenland white‐fronted geese decreased ~50% from 35,000 individuals in 1999 to 18,000 in 2022 (Fox et al., 2023), although the demographic mechanisms, associated environmental drivers and source‐sink dynamics contributing to this decline remain unclear.

Previous demographic analyses relied primarily on individuals captured at Wexford, the largest subpopulation during winter (Weegman et al., 2016, 2022). From 1983 to 2010, low and declining fecundity contributed more to subpopulation change at Wexford than survival, which remained relatively stable (Weegman et al., 2016). Current conservation plans assume this pattern applies to other subpopulations during winter, but we anticipate differences in environmental conditions throughout the full annual cycle may cause demographic heterogeneity. Although census data suggested stable abundance at Wexford, an IPM combining census data with capture–recapture and fecundity information revealed Wexford as a sink, with mortality exceeding recruitment, and abundance was primarily sustained by a high immigration rate (Weegman et al., 2016). Extending this analysis to more subpopulations to identify sources and sinks will improve our understanding of abundance trends and maximise conservation efforts to safeguard the entire Greenland white‐fronted goose metapopulation.

Recent low fecundity in Greenland white‐fronted geese may result from a ‘phenological mismatch’ between spring migration and breeding caused by climate change accelerating spring phenology. Greenland white‐fronted geese advanced their departures from wintering to spring staging areas by 15 days from 1969 to 2012 (Fox et al., 2014). Mismatches between plant nutrients and migration timing may reduce fecundity, as feeding and energy acquisition throughout spring migration are important for breeding success of this species (Cunningham et al., 2023; Schindler et al., 2024). Greenland white‐fronted geese did not shift arrival time on breeding areas from 1997 to 2013 (Fox et al., 2014), and increased spring snowfall in west Greenland (due to changes in the North Atlantic Oscillation) is thought to have reduced foraging opportunities after arrival, reducing breeding success (Boyd & Fox, 2008; Weegman et al., 2017). However, more research is needed to quantify the effects of these changing conditions on fecundity at subpopulation levels.

While Greenland white‐fronted goose survival is generally high and stable (Weegman et al., 2016, 2022), hunting and environmental conditions may cause heterogeneity in survival among subpopulations. Greenland white‐fronted geese have been protected from hunting since 1982 in Ireland and Scotland, 2006 in Iceland and 2009 in Greenland (Stroud et al., 2012). However, the impacts of residual illegal hunting pressure on survival and its variation among subpopulations remain uncertain. Severe storms during migration also greatly increase energy expenditure and pose a significant mortality risk to many migratory bird species, especially when migrations include crossing large water bodies such as oceans (Newton, 2024; Pennycuick & Battley, 2003; Tavares et al., 2020).

We developed an IMM spanning winter 1983/1984 to 2021/2022, incorporating annual capture–resighting, fecundity, subpopulation size and environmental information. Our objectives were to (1) estimate survival, fecundity, immigration and emigration rates for three primary subpopulations during winter (comprising ~63% of the annual metapopulation abundance) and a combined ‘Elsewhere’ set of subpopulations during winter (smaller subpopulations comprised the remaining ~37% of the metapopulation), (2) test hypotheses about how changing hunting and weather conditions throughout the annual cycle affected annual survival and fecundity, (3) determine the contributions of demographic rates to subpopulation growth rates and (4) identify whether each subpopulation group functioned as a source or a sink. These analyses aim to determine the demographic mechanisms and environmental drivers behind the persistent decline in this metapopulation, demonstrate the metapopulation source‐sink dynamics, and inform conservation strategies to maximise range‐wide abundance. This study emphasises the importance of quantifying subpopulation‐metapopulation dynamics to improve our understanding of population ecology and conservation management of fragmented animal populations.

## 2 METHODS

### 2.1 Study area

**FIGURE 1:**
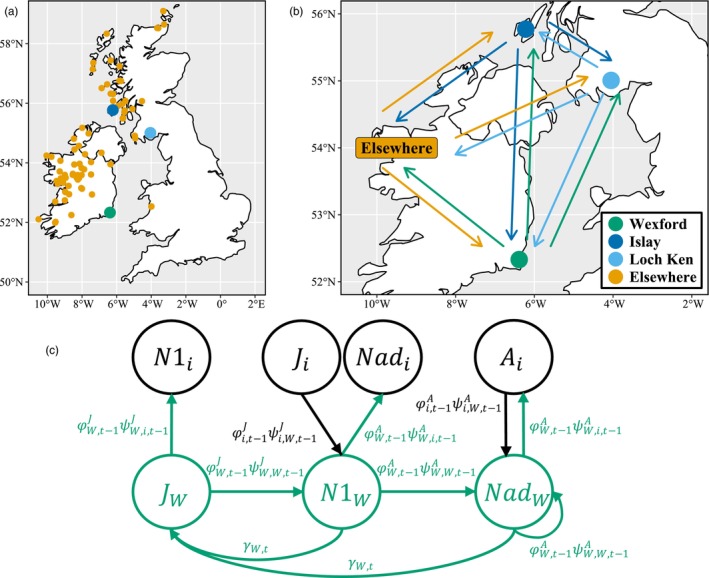
(a) Distribution of Greenland white‐fronted goose wintering subpopulations in Great Britain and Ireland, including three focal subpopulations used in analyses (Wexford: Green, Islay: Dark blue, Loch Ken: Light blue) and 65 subpopulations grouped into an ‘Elsewhere’ category (yellow). (b) Illustration of all annual movements among wintering subpopulations estimated in the integrated metapopulation model. Points represent focal subpopulations and Elsewhere collectively represents non‐focal subpopulations. (c) Life cycle diagram of a single Greenland white‐fronted goose subpopulation (Wexford [*W*], but similarly applicable to all subpopulations) with two adult stages: One‐year olds (N1; i.e. juveniles [J] from the previous year that survived and returned/moved to Wexford) and 2+‐year olds (Nad; i.e., N1 and Nad from the previous year that survived and returned/moved to Wexford). We estimated all demographic parameters, including stage‐specific survival ($\varphi^{J},\varphi^{A}$), per‐capita production of young (γ), and movement among each pair of subpopulations ($\psi^{J},\psi^{A}$) as time dependent. For simplicity, movement is here depicted between Wexford (green) and one other subpopulation (*i*; black), but we similarly estimated movement among all four subpopulation groups (see (b) for all possible movements).

During winters 1983/1984–2021/2022, Greenland white‐fronted geese aggregated at 68 known discrete wintering sites (which we here define as subpopulations) across Great Britain and Ireland (Fox et al., 1998; Ruttledge & Ogilvie, 1979). We studied the demography of geese wintering at Wexford Slobs, Ireland (52°21′ N 06°24′ W), Islay, Scotland (55°47′ N 06°15′ W), and Loch Ken, Scotland (55°00′ N 04°01′ W; Figure 1a) from winter 1983/1984 to winter 2021/2022. We define Greenland white‐fronted goose metapopulations based on wintering distribution but note that geese from these three focal subpopulations demonstrated high migratory connectivity throughout staging and breeding periods as well (Figures S1–S5). We combined information from the 65 other subpopulations during winter into a single ‘Elsewhere’ category as we lacked sufficient data for these to estimate subpopulation‐specific demographic parameters.

### 2.2 Data sources

#### 2.2.1 Fecundity

A coordinated observer network sampled annual age ratios (i.e. the ratio of juvenile [first winter] to adult [second winter and older] geese) at each wintering subpopulation during our study period, from which we generated the number of adults and juveniles each year at each subpopulation. Numbers of juveniles in wintering subpopulations therefore postdate mortality during hatching, brood‐rearing, fledging and migration prior to age ratio sampling. Numbers of adults and juveniles Elsewhere were based on a weighted average (by total subpopulation count) of age ratios among the Elsewhere subpopulations.

#### 2.2.2 Capture–mark–resighting

Among focal subpopulations and study years, we cannon‐netted 1061 juvenile and 1153 adult Greenland white‐fronted geese (with approximately even numbers of males and females) during winter. We did not have enough juvenile geese captured Elsewhere to include in our analysis. Captured geese were assigned an age class (juvenile or adult) by plumage characteristics (Cramp & Simmons, 1977), sexed by cloacal examination (Warren et al., 1992), and each marked with a metal leg band, white plastic leg band and orange neck collar (plastic band and collar inscribed with identical alphanumeric codes, collar codes legible at up to 800 m using 20–60× spotting scopes; Warren et al., 1992; Weegman et al., 2016). An observer network resighted geese annually at focal subpopulations and Elsewhere, generating 53,367 goose resightings during our study period. Captures and handling of wild geese were permitted through the Icelandic Institute of Natural History, National Parks and Wildlife Service (Ireland) and British Trust for Ornithology.

#### 2.2.3 Population surveys

Observers counted Greenland white‐fronted geese annually at each subpopulation during 26 March–10 April (Fox et al., 1998). When counts were missing, we used linear interpolation to fill missing data (see Supporting Information; Table S1, for additional details). We summed annual counts among all non‐focal subpopulations to comprise Elsewhere. Given the even sex ratio among Greenland white‐fronted geese (Weegman et al., 2015), we halved counts and based analyses on females only.

#### 2.2.4 Weather

We combined contemporary GPS tracking data (Ozsanlav‐Harris, 2023; Schindler et al., 2024) with historical breeding season aerial survey data (Malecki et al., 2000), staging area collar resighting data (Fox et al., 1983; Francis & Fox, 1987) and wintering census data (Fox et al., 1998) to delineate areas used by each focal subpopulation and Elsewhere during breeding, migration, staging, and winter phases of the annual cycle, and used recorded observations and GPS tracking data to determine timing of these phases (see Supporting Information; Figures S1–S5). We obtained hourly surface‐level temperature and barometric pressure data from the ERA5 dataset (30‐m resolution; Hersbach et al., 2023) and daily cumulative precipitation from the Climate Prediction Center Global Unified Precipitation dataset (0.5° latitude × 0.5° longitude resolution; Xie et al., 2007), and used these data to calculate cumulative growing degree days (GDD; i.e. a proxy for vegetation phenology) on wintering, spring staging, and breeding areas, cumulative snowfall on breeding areas, and number of severe storms during migration (Figure S6; see Supporting Information for details).

### 2.3 Modelling approach

We developed a female‐based IMM to describe spatiotemporal differences in Greenland white‐fronted goose fecundity and juvenile‐ and adult‐specific survival and dispersal for our three focal subpopulations and Elsewhere from winter 1983/1984 to 2021/2022 (Figure 1b,c). We also quantified weather effects on fecundity and both weather and hunting on survival. Our IMM comprised a population model linking population size with demographic rates, and component likelihoods of the three data sets (i.e. sub‐models): (1) count sub‐model, (2) fecundity sub‐model and (3) survival and dispersal sub‐model.

#### 2.3.1 Population model

We specified total subpopulation size (${\text{Ntot}}_{i,t}$) of females in subpopulation *i* in year *t* as a sum of the number of female juveniles that hatched and survived to winter ($J_{i,t}$), number of female juveniles that survived from first to second winter and returned as adults ($N1_{i,t}$), number of female adults that survived from second (or older) to subsequent winter and returned (${\mathrm{Nad}}_{i,t}$) and number of female immigrants (second winter or older) from other subpopulations ($I_{i,t}$). For example, we specified subpopulation size at Wexford (*W*) as

$$J_{W,t}\sim \text{Poisson}\left(A_{W,t}\gamma_{W,t}\right),$$

$$N1_{W,t}\sim \text{Binomial}\left(\varphi_{W,t-1}^{J}\left(1-\psi_{W,\text{Is},t-1}^{J}+\psi_{W,\mathrm{LK},t-1}^{J}+\psi_{W,E,t-1}^{J}\right),J_{W,t-1}\right),$$

$${\mathrm{Nad}}_{W,t}\sim \text{Binomial}\left(\varphi_{W,t-1}^{A}\left(1-\psi_{W,\text{Is},t-1}^{A}+\psi_{W,\mathrm{LK},t-1}^{A}+\psi_{W,E,t-1}^{A}\right),A_{W,t-1}\right),$$

$$\begin{array}{c} I_{W,t}\sim \text{Poisson}\left(\varphi_{\text{Is},t-1}^{J}\right.\psi_{\text{Is},W,t-1}^{J}J_{\text{Is},t-1}+\varphi_{\mathrm{LK},t-1}^{J}\psi_{\mathrm{LK},W,t-1}^{J}\;J_{\mathrm{LK},t-1}+\varphi_{\text{Is},t-1}^{A}\psi_{\text{Is},W,t-1}^{A}\\ A_{\text{Is},t-1}+\varphi_{\mathrm{LK},t-1}^{A}\psi_{\mathrm{LK},W,t-1}^{A}A_{\mathrm{LK},t-1}+\varphi_{E,t-1}^{A}\left.\psi_{E,W,t-1}^{A},A_{E,t-1}\right), \end{array}$$

where $\gamma_{W,t}$ was fecundity at Wexford (i.e. number of juveniles recruited in year *t* to Wexford per adult at Wexford in year *t*), $\varphi_{W,t-1}^{J}$ and $\varphi_{W,t-1}^{A}$ were the probabilities a juvenile or adult, respectively, at Wexford in winter of year *t* − 1 survived to winter in *t*, $\psi_{i,W,t-1}^{J}$ and $\psi_{i,W,t-1}^{A}$ for all other subpopulations *i* (i.e. Islay [*Is*], Loch Ken [*LK*], Elsewhere [*E*]) were the probabilities a goose in the respective age class and subpopulation in year *t* − 1 moved to Wexford in year *t*. Thus, total number of female adults (A; i.e., second winter or older geese) was the sum of N1, Nad and I and Ntot the sum of J and A. We assumed no juvenile movement from Elsewhere to our focal subpopulations (e.g., assumed $\psi_{E,W}^{J}=0$) as we did not have sufficient capture history data for juveniles Elsewhere.

#### 2.3.2 Count sub‐model

We estimated annual subpopulation size with a state‐space model, with a state process model as defined in the population model. In the observation model, we linked true subpopulation size to subpopulation count (y) by:

$$y_{i,t}\sim \text{logNormal}\left(\log\left({\text{Ntot}}_{i,t}\right),\tau^{y}\right).$$

#### 2.3.3 Fecundity sub‐model

We considered fecundity for subpopulation *i* in year *t* as a function of GDD during early spring on wintering areas (${\mathrm{GDD}}^{\text{winter}}$), during spring on staging areas (${\mathrm{GDD}}^{\text{spring}}$) and during late spring/early summer on breeding areas (${\mathrm{GDD}}^{\text{breed}}$), and cumulative snow (${\text{snow}}^{\text{breed}}$) throughout winter and spring on breeding areas such that:

$$\begin{array}{c} \log\left(\gamma_{i,t}\right)=\alpha_{i}^{\gamma }+\beta_{1,i}{\mathrm{GDD}}_{i,t}^{\text{winter}}+\beta_{2,i}{\mathrm{GDD}}_{i,t}^{\text{stage}}+\beta_{3,i}{\mathrm{GDD}}_{i,t}^{\text{breed}}\\ +\beta_{4,i}{\text{snow}}_{i,t}^{\text{breed}}+\varepsilon_{i,t}^{\gamma }, \end{array}$$

where $\alpha^{\gamma }$ was among‐year mean per‐capita production of young, $\varepsilon^{\gamma }$ were process errors that followed Normal distributions with mean 0 and standard deviation $\sigma^{\gamma }$. We estimated annual fecundity with a state‐space model, with a state process model as defined in the population model. In the observation model, we linked true number of juveniles in each wintering subpopulation to number of juveniles counted in the corresponding wintering subpopulation ($J^{\mathrm{obs}}$) by:

$$J_{i,t}^{\mathrm{obs}}\sim \text{Poisson}\left(J_{i,t}\right).$$

#### 2.3.4 Survival and dispersal sub‐model

We considered juvenile and adult survival as a function of cumulative number of severe storms during spring and autumn migration (storms) and hunting protection (hunt) such that:

$$\text{logit}\left(\varphi_{i,t}^{J}\right)=\alpha_{i}^{\mathrm{\varphi J}}+\beta_{5,i}{\text{storms}}_{i,t}+{\text{hunt}}_{1,i,h}+\varepsilon_{i,t}^{\mathrm{\varphi J}},$$

$$\text{logit}\left(\varphi_{i,t}^{A}\right)=\alpha_{i}^{\mathrm{\varphi A}}+\beta_{6,i}{\text{storms}}_{i,t}+{\text{hunt}}_{2,i,h}+\varepsilon_{i,t}^{\mathrm{\varphi A}},$$

where $\alpha^{\mathrm{\varphi J}}$ and $\alpha^{\mathrm{\varphi A}}$ were among‐year mean juvenile and adult survival, respectively, and $\varepsilon^{\mathrm{\varphi J}}$ and $\varepsilon^{\mathrm{\varphi A}}$ were process errors that followed Normal distributions with mean 0 and standard deviations $\sigma^{\mathrm{\varphi J}}$ and $\sigma^{\mathrm{\varphi A}}$, respectively. Hunting protections were specified as intercepts for level of hunting protection *h* (i.e. additional protection in Iceland [2006–2008] or in both Iceland and Greenland [2009–2022] with respect to protection in Ireland and Scotland only [1983–2005]). We specified subpopulation‐specific β and hunt shrinkage priors as:

$$\beta_{i}\sim \text{Laplace}\left(\mu^{\beta },1/{\lambda^{\beta }}^{2}\right),$$

$${\text{hunt}}_{i,h}\sim \text{Laplace}\left(\mu_{h}^{\text{hunt}},1/{\lambda_{h}^{\text{hunt}}}^{2}\right).$$

This parameterisation helped shrink subpopulation‐specific β and hunt towards among‐subpopulation means ($\mu^{\beta }$) when sparsity of capture–recapture data for Loch Ken limited our ability to detect annual variation in survival for this subpopulation.

We estimated survival, movement and resighting (*p*) probabilities using a multistate capture–recapture model with a multinomial likelihood (see Supporting Information for details). We specified all movement and resighting probabilities as time‐dependent with random year ($\varepsilon^{\psi }$ and $\varepsilon^{p}$) deviations from among‐year means ($\alpha^{\psi }$ and $\alpha^{p}$) that followed Normal distributions with mean 0 and standard deviations $\sigma^{\psi }$ and $\sigma^{p}$, respectively. Because we did not have enough juvenile geese captured Elsewhere to include in our analysis, we only estimated adult survival and movement probabilities for the Elsewhere group.

### 2.4 Model implementation

We implemented the IMM in a hierarchical Bayesian framework with posterior distributions obtained by Markov Chain Monte Carlo using NIMBLE version 1.2.0 (de Valpine et al., 2017; NIMBLE Development Team, 2023) in R (R Development Core Team, 2023). We assumed $A_{i,1}\sim \text{DiscreteUniform}\left(1000,4000\right)$ for Wexford and Islay, $A_{\mathrm{LK},1}\sim \text{DiscreteUniform}\left(1,400\right)$, $A_{E,t}\sim \text{DiscreteUniform}\left(1000,7500\right)$, $\alpha_{i}^{\gamma }\sim \mathrm{Uniform}\left(0,1\right)$, $\sigma^{\gamma }\sim \text{Uniform}\left(0,10\right)$, $\beta_{1-4,i}\sim \text{Normal}\left(0,10\right)$, $\mu^{\beta }\sim \text{Normal}\left(0,1\right)$, $\lambda^{\beta }\sim \text{Exponential}\left(0.1\right)$, $\mu^{\text{hunt}}\sim \text{Normal}\left(0,1\right),$ $\lambda_{h}^{\text{hunt}}\sim \text{Exponential}\left(0.1\right)$, $\alpha_{i}^{p}\sim \text{Uniform}\left(0,1\right)$, $\sigma^{p}\sim \text{Uniform}\left(0,10\right)$, and $\tau^{y}\sim \text{InverseGamma}\left(0.01,0.01\right)$. We assumed $\alpha^{\psi }\sim \text{Uniform}\left(0,0.2\right)$ and $\sigma^{\psi }\sim \text{Uniform}\left(0,1\right)$ for all dispersal probabilities (movement among subpopulations, e.g., $\psi_{\text{Is},W,t-1}^{J}$). We used three chains, each with 270,000 iterations including 10,000 burn‐in and thinned by 10, yielding 78,000 posterior samples for each parameter. We assessed convergence using the Gelman–Rubin statistic ($\hat{R}<1.1$; Brooks & Gelman, 1998) and visual inspection of traceplots. Integrated population models rely on a range of simplifying assumptions for each sub‐model and for integrating the component data likelihoods to form the joint likelihood (see Schaub & Kéry, 2021; Weegman et al., 2021 for full reviews). We used posterior predictive checks to assess assumption violations and check the fit of our fecundity, survival and dispersal and population sub‐models (Schaub & Kéry, 2021; Frost et al., 2023; Christian et al., 2024; see Supporting Information for details). We report the proportion of the posterior distribution above or below 0 on the same side as the mean for each environmental covariate β and hunt intercept as evidence that covariate effects were positive or negative. We considered more than 70% of the posterior distribution above or below 0 as moderate support and more than 90% as strong support.

### 2.5 Demographic contributions to subpopulation growth rate

To retrospectively assess contributions of demographic rates and population structure to subpopulation change, we used transient life‐table response experiments (tLTREs; Koons et al., 2016, 2017) to decompose the temporal variance of the realised subpopulation growth rates ($\lambda_{i,t}={\text{Ntot}}_{i,t+1}/{\text{Ntot}}_{i,t}$) due to the realised temporal variation in demographic parameters ($\theta_{k}$, where *k* included fecundity, age‐ and subpopulation‐specific survival and immigration rates, and the proportional abundance of each stage class [J, N1, Nad and I]). See Supporting Information for full parameterisation of growth rate expressions. The extent to which $\lambda_{i,t}$ was sensitive ($\frac{\delta \lambda_{i,t}}{\delta \theta_{i,k,t}}$) to changes (δ) in $\theta_{i,k,t}$ was used to estimate the amount that variation in each $\theta_{i,k,t}$ contributed to temporal variation in $\lambda_{i,t}$:

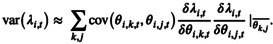




We also calculated year‐to‐year changes in the observed contributions of $\theta_{i,k,t}$ to sequential changes in $\lambda_{i,t}$ ($∆\lambda_{i,t}$) to assess temporal patterns in the demographic contributions to $∆\lambda_{i,t}$:

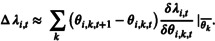




We did not evaluate tLTREs for Elsewhere as we did not estimate Elsewhere juvenile survival or movement probabilities.

### 2.6 Source‐sink dynamics

To determine whether subpopulations functioned as sources (i.e. emigration > immigration) or sinks (i.e. emigration < immigration), we calculated post hoc net immigration rates for adult geese for each subpopulation group (see Supporting Information for details). We additionally formed post hoc population projection matrices (Carslake et al., 2009; Stott et al., 2011; Weegman et al., 2016) for each focal subpopulation. We used the posterior distributions for age‐specific survival, age‐specific movement probabilities, and recruitment to predict annual subpopulation size and growth rates for each focal subpopulation for four scenarios: (1) emigration but no immigration included, (2) immigration but no emigration included, (3) no emigration or immigration included and (4) both immigration and emigration included. We compared the posterior distributions for total subpopulation size and among‐year mean growth rates from each scenario to assess the relative contributions of emigration and immigration to subpopulation growth rate.

## 3 RESULTS

### 3.1 Model fit

Posterior predictive checks indicated that the model fit the data well overall. Bayesian *p*‐values for the fecundity sub‐model were 0.51–0.66 and for the population sub‐model were 0.25–0.68 (Figures S7–S10). For the survival and dispersal sub‐model, Bayesian *p*‐values were 0.25 for combined detections (Figure S11), 0.14 for Wexford detections and 0.62 for Elsewhere detections (Figures S12a,d and S13a,d). We found some evidence for lack of fit for Islay and Loch Ken detections (Bayesian *p*‐values of 0.00 and 0.01, respectively; Figure S13b,c). Graphical tools illustrated the potential for our model to overestimate detections for Islay early in the study period and underestimate detections for Loch Ken late in the study period, likely due to smaller sample sizes of marked geese during these intervals (Figure S12b,c).

### 3.2 Demographic estimates

**FIGURE 2:**
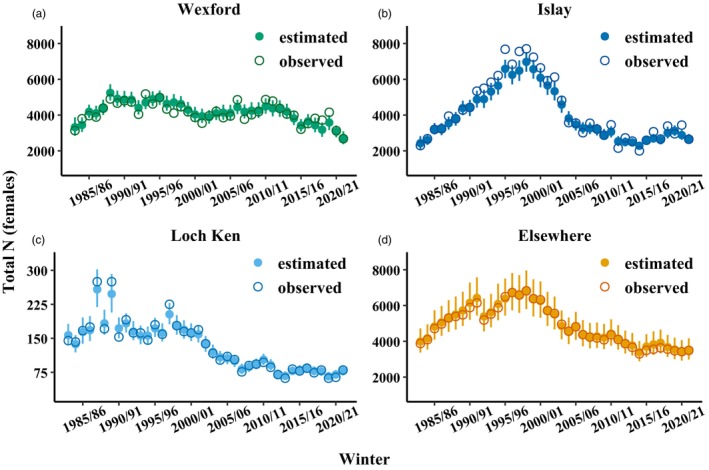
Annual number of Greenland white‐fronted goose females in the (a) Wexford, (b) Islay, (c) Loch Ken and (d) ‘Elsewhere’ subpopulation groups. Open circles represent number of observed females (i.e. count data) and filled circles represent estimated number of females from the integrated metapopulation model (posterior medians with 90% credible intervals).

The Wexford subpopulation contained 3320 total females (90% credible interval [CRI] = 2845–3875) in winter 1983/1984. Annual subpopulation growth rates for Wexford varied among years from 0.86 (90% CRI = 0.77–0.97) to 1.21 (90% CRI = 1.10–1.36) but were generally <1 after 2010 as the subpopulation declined to 2717 females (90% CRI = 2372–3093) in winter 2021/2022 (Figure 2a; Figure S14a). Islay contained 2428 (90% CRI = 2102–2817) total females in winter 1983/1984. Islay growth rates ranged from 0.83 (90% CRI = 0.73–0.93) to 1.23 (90% CRI = 1.10–1.38), were generally >1 until the subpopulation peaked at 6974 (90% CRI = 6422–7524) females in winter 1998/1999, and generally <1 afterwards as the subpopulation declined to 2660 (90% CRI = 2399–2941) females in winter 2021/2022 (Figure 2b; Figure S14b). Loch Ken contained 157 (90% CRI = 135–182) females in winter 1983/1984. Loch Ken growth rates ranged from 0.69 (90% CRI = 0.57–0.84) to 1.53 (90% CRI = 1.20–1.92), were variable before 1997/1998 and generally <1 afterwards as the subpopulation declined to 78 females (90% CRI = 68–90) in winter 2021/2022 (Figure 2c; Figure S14c). There were 3987 females in the Elsewhere group in 1983/1984. Annual subpopulation growth rates for Elsewhere ranged from 0.84 (90% CRI = 0.66–1.07) to 1.16 (90% CRI = 0.91–1.47), were generally >1 until a peak of 6818 females (90% CRI = 5802–7959) in winter 1998/1999 and <1 afterwards as numbers declined to 3521 females (90% CRI = 2978–4169) (Figure 2d; Figure S14d).

**FIGURE 3:**
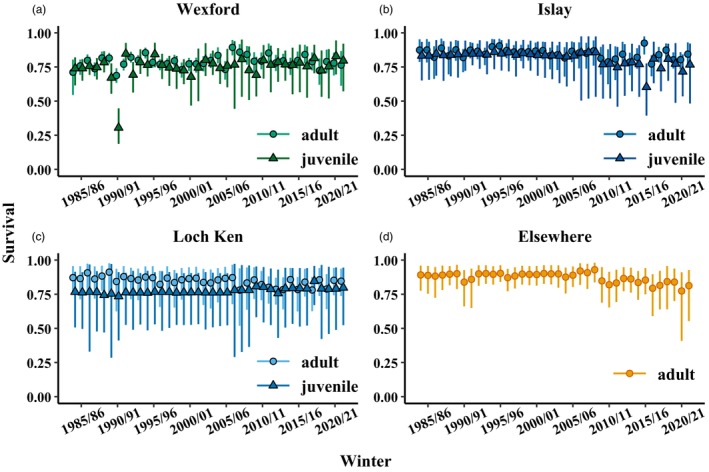
Annual stage‐specific survival estimates (i.e. survival from winter *t* − 1 to winter *t*; posterior medians with 90% credible intervals) for marked Greenland white‐fronted geese (of both sexes) in the (a) Wexford, (b) Islay, (c) Loch Ken and (d) ‘Elsewhere’ subpopulation groups. We considered juvenile survival as the period from first to second winter (represented by triangles) and adult survival as the period from the second or a later winter to subsequent winters (circles).

Annual juvenile survival was lower than adults among all subpopulation groups, but survival of both age classes was relatively stable (Figure 3). Wexford geese had the lowest mean survival (juvenile: 0.74, 90% CRI = 0.70–0.79; adult: 0.79, 90% CRI = 0.76–0.81; Figure 3a), followed by Loch Ken (juvenile: 0.76, 90% CRI = 0.66–0.84; adult: 0.86, 90% CRI = 0.79–0.91; Figure 3c), Islay (juvenile: 0.84, 90% CRI = 0.79–0.85; adult: 0.86, 90% CRI = 0.83–0.89; Figure 3b) and Elsewhere geese (adult: 0.89, 90% CRI = 0.86–0.92; Figure 3d). Resighting probability for marked geese in focal subpopulations was high (Wexford: 0.90, 90% CRI = 0.85–0.93; Islay: 0.89, 90% CRI = 0.84–0.93; Loch Ken: 0.92, 90% CRI = 0.84–0.97) but low for geese Elsewhere (0.28, 90% CRI = 0.24–0.33; Figure S15).

**FIGURE 4:**
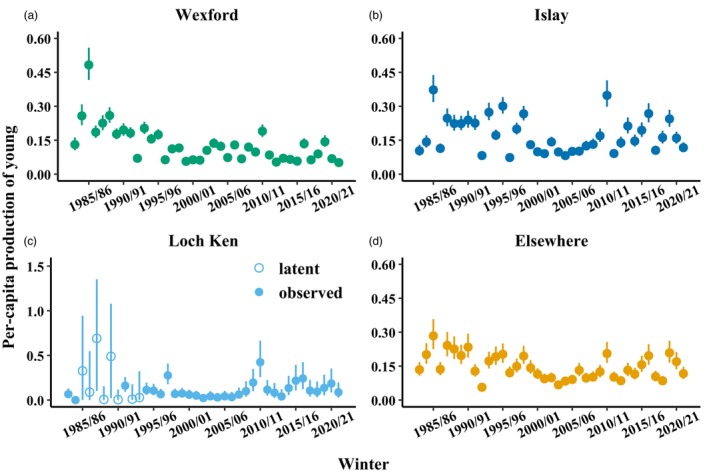
Annual per‐capita production of young estimates (posterior medians with 90% credible intervals) for Greenland white‐fronted geese in the (a) Wexford, (b) Islay, (c) Loch Ken and (d) ‘Elsewhere’ subpopulation groups. Open circles represent occasions when per‐capita production of young was latent (i.e. no fecundity data existed).

Mean per‐capita production of young was low for all subpopulation groups throughout the study period (Figure 4), lowest at Loch Ken (0.10, 95% CRI = 0.03–0.40; Figure 4c) followed by Wexford (0.11, 90% CRI = 0.05–0.26; Figure 4a), Elsewhere (0.14, 90% CRI = 0.11–0.20; Figure 4d), and Islay (0.15, 95% CRI = 0.09–0.31; Figure 4b).

### 3.3 Environmental and hunting drivers of demography

**FIGURE 5:**
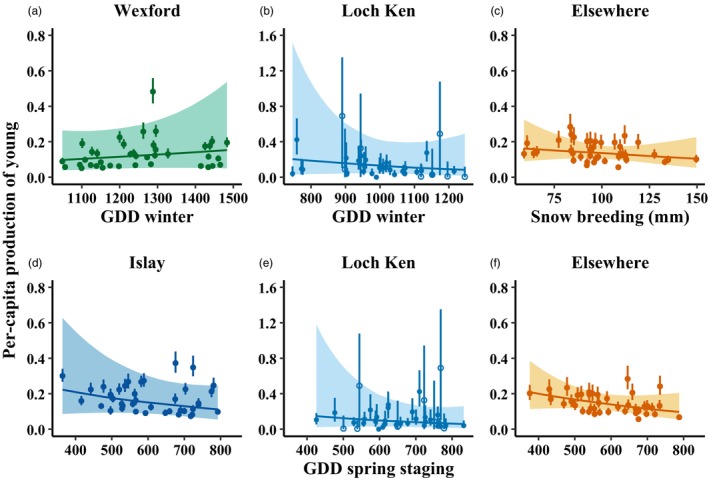
Response curves showing the effects of (a, b) growing degree days on wintering areas, (c) cumulative snow on breeding areas, and (d–f) growing degree days on spring staging areas on per‐capita production of young in Greenland white‐fronted geese. Lines depict predicted relationships, shaded areas depict prediction uncertainty (90% credible intervals) and points (with 90% credible interval error bars) depict the estimated per‐capita production of young. Open circle points designate per‐capita production of young estimates from years without fecundity data.

GDD on wintering areas positively affected per‐capita production of young at Wexford (β = 0.16, 90% CRI = −0.29–0.74, Pr(β > 0) = 0.74) and negatively at Loch Ken (β = −0.26, 90% CRI = −1.15–0.58, Pr(β < 0) = 0.71; Figure 5a,b; Figure S16). GDD on spring staging areas negatively affected per‐capita production of young at Islay (β = −0.19, 90% CRI = −0.49–0.15, Pr(β < 0) = 0.83), Loch Ken (β = −0.27, 90% CRI = −0.98–0.42, Pr(β < 0) = 0.76), and Elsewhere (β = −0.21, 90% CRI = −0.49–0.11, Pr(β < 0) = 0.87; Figure 5d–f; Figure S16). Cumulative snow on breeding areas negatively affected per‐capita production of young Elsewhere (β = −0.15, 90% CRI = −0.56–0.27, Pr(β < 0) = 0.74; Figure 5c; Figure S16). GDD on breeding areas had no strong effect on per capita production of young of any subpopulation group (Figure S16). Compared to years with hunting protection in Ireland and Great Britain only, adult survival was higher following additional hunting protection in Iceland for Wexford (β=0.43, 90% CRI = −0.08–0.98, Pr(β > 0) = 0.92) and Elsewhere geese (β=0.27, 90% CRI = −0.42–1.21, Pr(β > 0) = 0.73; Figure S17a). After additional hunting protection in Greenland, juvenile survival was higher for Wexford geese (β=0.16, 90% CRI = −0.33–0.70, Pr(β > 0) = 0.70) but lower for Islay geese (β = −0.38, 90% CRI = −1.08–0.26, Pr(β < 0) = 0.83), and adult survival was lower for Islay (β=−0.29, 90% CRI = −0.61–0.03, Pr(β < 0) = 0.93), Loch Ken (β=−0.34, 90% CRI = −0.83–0.08, Pr(β < 0) = 0.91), and Elsewhere geese (β = −0.90, 90% CRI = −0.90–−0.03, Pr(β < 0) = 0.96; Figure S17a). Storm days negatively affected juvenile survival at Wexford (β=−0.14, 90% CRI = −0.35–0.09, Pr(β < 0) = 0.85), but positively affected adult survival at Wexford (β = 0.07, 90% CRI = −0.07–0.20, Pr(β > 0) = 0.80) and Loch Ken (β=0.12, 90% CRI = −0.15–0.42, Pr(β > 0) = 0.76; Figure S17b).

### 3.4 Demographic contributions to subpopulation growth rate

Among all years at Wexford, fluctuations in adult immigration from Elsewhere (40.3% of total variation, 90% CRI = 20.8%–59.8%) and fecundity (37.9%, 90% CRI = 23.4%–54.2%) contributed most to temporal variation in realised subpopulation growth rates, followed by adult survival (12.0%, 90% CRI = 0.0%–25.3%) and adult immigration from Islay (10.0%, 90% CRI = 2.3%–20.9%; Figure S18a). Population growth rates at Wexford were insensitive to all other demographic parameters and population structure. Adult immigration from Elsewhere was the dominant driver in 41.7% of study years, fecundity in 30.6% of study years, and adult survival in 25.0% of years (Figure S19). In successive years when population growth rates changed by large amounts (∆>0.1 or ∆<0.1), the dominant driver was typically either adult immigration (44.4% of cases) or fecundity (44.4%; Figure S19).

At Islay, fluctuations in fecundity (32.7% of total variation, 90% CRI = 21.6%–44.8%), adult immigration from Elsewhere (29.3% of total variation, 90% CRI = 10.5%–49.3%), and adult survival (22.4% of total variation, 90% CRI = 10.1%–36.1%) contributed most to temporal variation in realised population growth rates, while adult immigration from Wexford (5.8%, 90% CRI = 2.0%–10.8%; Figure S18b) contributed a small amount. Population growth rates at Islay were insensitive to all other demographic parameters and population structure. Fecundity was the dominant driver in 47.2% of years, adult survival in 30.6% of years, adult immigration from Wexford in 11.1% of years and adult fidelity in 8.3% of years (Figure S20). In successive years when population growth rates changed by large amounts, the dominant driver was typically fecundity (60.0% of cases; Figure S20). During the first half of the study period, the dominant driver was typically adult survival (44.4% of 18 years), while fecundity was typically the dominant driver during the second half of the study period (66.7% of 18 years), corresponding with periods of lower fecundity and population growth rates typically <1 (Figure S20).

Among all years at Loch Ken, fluctuations in fecundity (58.3% of total variation, 90% CRI = 30.8%–82.5%) contributed most to temporal variation in realised population growth rates, while adult survival (8.4%, 90% CRI = 0.0%–20.3%), proportion of juveniles (7.2%, 90% CRI = 0.0%–16.1%), adult fidelity (7.0%, 90% CRI = 0.0%–18.4%), adult immigration from Elsewhere (6.3%, 90% CRI = 0.0%–27.2%), adult immigration from Wexford (4.1%, 90% CRI = 0.0%–18.4%) and adult immigration from Islay (3.2%, 90% CRI = 0.0%–17.2%; Figure S18c) contributed a small amount. Population growth rates at Loch Ken were insensitive to all other demographic parameters and population structure. Fecundity was the dominant driver in 50.0% of years, adult survival in 25.0% of years, and adult immigration from Elsewhere in 11.1% of years (Figure S21). In successive years when population growth rates changed by large amounts, the dominant driver was typically either fecundity (54.5% of cases) or adult survival (22.7%; Figure S21). During the first half of the study period, the dominant driver was typically fecundity (66.7% of 18 years), corresponding with years when fecundity was highly variable, while adult survival and fecundity were both commonly dominant drivers during the second half of the study period (44.4% and 33.3% of 18 years, respectively), corresponding with periods of low fecundity, increased variability in adult survival, and population growth rates typically <1 (Figure S21).

### 3.5 Source‐sink dynamics

**FIGURE 6:**
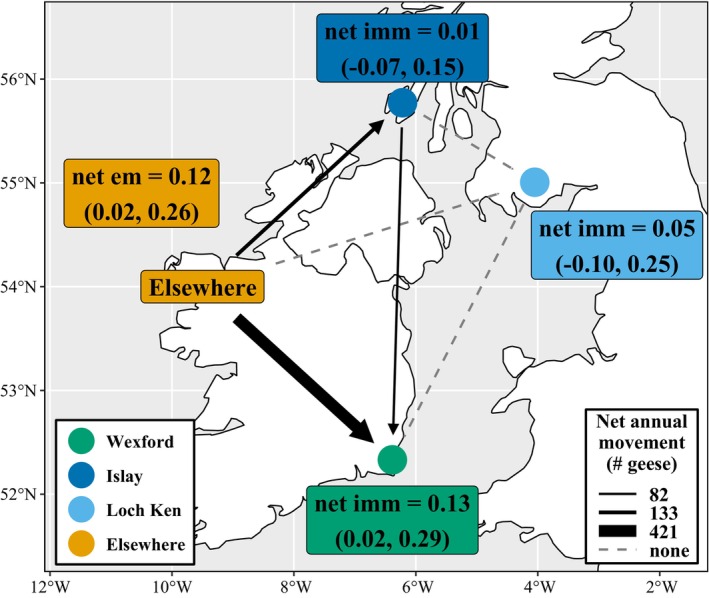
Net immigration (i.e. immigration > emigration) or emigration (i.e. emigration > immigration) rates among Greenland white‐fronted goose wintering subpopulations (90% credible intervals shown in parentheses). Points represent focal subpopulations and ‘Elsewhere’ collectively represents non‐focal subpopulations. Arrows illustrate the average net number (arrow size) and direction of geese that moved annually among subpopulations. Dashed lines represent no net movement (i.e. immigration rates typically balanced by similar emigration rates). Note that net immigration rates were calculated for adults only to allow comparison among focal subpopulation groups and Elsewhere.

Wexford had an annual net immigration rate of 0.13 (90% CRI = 0.02–0.29; Figure 6), primarily from Elsewhere ($I^{\mathrm{net}}$ = 0.11, 90% CRI = 0.01–0.26), and to a lesser extent, Islay ($I^{\mathrm{net}}$ = 0.01, 90% CRI = −0.03–0.09; Figure 6). Islay received net immigrants from Elsewhere ($I^{\mathrm{net}}$ = 0.03, 90% CRI = −0.03–0.17), but lost net emigrants to Wexford ($I^{\mathrm{net}}$ = −0.02, 90% CRI = −0.09–0.03), resulting in no overall net immigration or emigration ($I^{\mathrm{net}}$ = 0.01, 90% CRI = −0.07–0.15; Figure 6). Loch Ken had no overall net immigration or emigration ($I^{\mathrm{net}}$ = 0.05, 95% CRI = −0.10–0.25), and no net immigration or emigration from any subpopulation (Figure 6). The Elsewhere subpopulation had annual net emigration ($I^{\mathrm{net}}$ = −0.12, 95% CRI = −0.26 to −0.02), resulting from net emigration to both Wexford ($I^{\mathrm{net}}$ = −0.09, 90% CRI = −0.22–0.01) and Islay ($I^{\mathrm{net}}$ = −0.02, 90% CRI = −0.11–0.02; Figure 6).

Projected among‐year Wexford subpopulation growth rates were similar when including immigration and emigration (0.99, 90% CRI = 0.99–1.00) or only immigration (1.01, 90% CRI = 1.01–1.02) in projection matrices, but were lower when no movement (0.88, 90% CRI = 0.87–0.89) or only emigration was included (0.77, 90% CRI = 0.76–0.78; Figure S22). Projected subpopulation size was also strongly correlated with estimated subpopulation size when including immigration and emigration (*r* = 0.99, Pr(r > 0) = 1.00) or only immigration (*r* = 0.76, Pr(r > 0) = 1.00), but less correlated when no movement (*r* = 0.42, Pr(r > 0) = 1.00) or only emigration (*r* = 0.13, Pr(r > 0) = 0.92) was included. For Islay and Loch Ken projection matrices, among‐year subpopulation growth rates were highest when only immigration was included (Islay: 1.04, 90% CRI = 1.03–1.05, Loch Ken: 1.02, 90% CRI = 1.00–1.04), similar when both immigration and emigration were included (Islay: 1.00, 90% CRI = 1.00–1.01, Loch Ken: 0.98, 90% CRI = 0.98–0.99) and no movement was included (Islay: 0.98, 90% CRI = 0.96–1.00; Loch Ken: 0.93, 90% CRI = 0.88–0.98), and lowest when only emigration was included (Islay: 0.87, 90% CRI = 0.85–0.88; Loch Ken: 0.75, 90% CRI = 0.70–0.80; Figure S22). Projected subpopulation size was also strongly correlated with estimated subpopulation size when both immigration and emigration was included (Islay: *r* = 1.00, Pr(r > 0) = 1.00; Loch Ken: *r* = 0.97, Pr(r > 0) = 1.00) or no movement was included (Islay: *r* = 0.90, Pr(r > 0) = 1.00; Loch Ken: *r* = 0.90, Pr(r > 0) = 1.00), but less strongly correlated when only immigration (Islay: *r* = 0.56, Pr(r > 0) = 1.00; Loch Ken: *r* = 0.54, Pr(r > 0) = 0.88) or emigration (Islay: *r* = 0.19, Pr(r > 0) = 0.99; Loch Ken: *r* = 0.59, Pr(r > 0) = 1.00) was included.

## 4 DISCUSSION

Using 39 years of demographic and environmental data in an integrated metapopulation model, we demonstrated how heterogeneity in survival, fecundity and movement rates among Greenland white‐fronted goose subpopulations contributed to observed changes in subpopulation abundance. We found that declines in abundance were strongly related to low reproductive rates. Changes in plant growth phenology throughout spring migration and snow on breeding areas appear to be reducing fecundity, though subpopulations have been differentially affected by these changes. While we found evidence of recent declines in abundance in all four subpopulation groups, asynchrony in timing of declines appears to be caused by differential immigration and emigration among subpopulation groups. Thus, only by estimating subpopulation‐specific survival and fecundity, and movement among subpopulations could we understand the demographic mechanisms and associated environmental drivers of observed changes in abundance.

Consistent with Weegman et al. (2016), Wexford functioned as a sink with low fecundity and survival rates, relative to other subpopulations, and was only maintained by high immigration (~13% of adults at Wexford) from Islay and Elsewhere. Contrary to previous assessments based on fewer years of data identifying Wexford abundance as stable, we found numbers there have declined since 2010, suggesting immigration can no longer maintain the subpopulation size because of simultaneously low fecundity. Smaller subpopulations in the Elsewhere group collectively functioned as a source, with high survival (relative to all focal subpopulations) and fecundity (relative to Wexford and Loch Ken). Their status as source subpopulations makes them important for conservation and range maintenance, even though the individual subpopulations comprising our Elsewhere category are much smaller than Wexford. Additionally, high emigration away from subpopulations in the Elsewhere group increases the risk of losing these subpopulations completely when fecundity is not high enough to balance net emigration; as a result, only 43 of the 65 Elsewhere subpopulations were observed persisting by the end of our study period. Due to limitations in our capture–mark–resighting data, we could not directly test hypothesised environmental drivers of movement among subpopulations. However, a separate analysis using GPS tracking data found that Greenland white‐fronted geese moved among wintering subpopulations based on locally available foraging conditions (Schindler et al., 2025), so uneven immigration/emigration rates may result from differences among subpopulations in foraging habitat quality. Immigration/emigration rates did not strongly affect trends in Islay or Loch Ken, but low fecundity has not consistently balanced mortality in either subpopulation since 1999, especially at Loch Ken.

Immigration and emigration processes contribute strongly to observed changes in abundance at small (e.g. subpopulation) scales in many animal populations (Millon et al., 2019; Schaub et al., 2013; van Oosten et al., 2015). Thus, it is important to account for these processes in population models. Many studies estimate immigration in IPMs as a ‘hidden’ parameter (i.e. not explicitly informed by data) through balancing estimates of population size, survival, and fecundity (Schaub & Kéry, 2021). While these immigration estimates are useful in many contexts and provide improved inference over models that do not account for immigration, these hidden parameters are sensitive to violations of model assumptions of any of the component datasets (Riecke et al., 2019), which may result in overestimating the contribution of immigration to population growth rates (Paquet et al., 2021). By incorporating explicit movement data in a multistate modelling framework, IMMs are less susceptible to these biases and provide an improved assessment of movement rates among subpopulations. While we found some evidence of potential biases in movement estimates for subpopulations and years with smaller capture–resighting data sample sizes, we found no strong contributions of movement to population growth rates in these instances, so the potential for these biases to affect our results was limited. Due to data limitations, we were also unable to estimate probabilities of juveniles moving from the Elsewhere group to our focal subpopulations; however, given the low fecundity and high site fidelity throughout the metapopulation (i.e. few moving juveniles with respect to overall subpopulation sizes), these movements had minimal capacity to affect subpopulation growth rates.

Migratory herbivores attempt to time spring migration to coincide with the peak spring growing season to ensure optimal plant nutritional quality along migratory routes (Merkle et al., 2016; van der Graaf et al., 2006; van Wijk et al., 2012). This may be especially important for Greenland white‐fronted geese because conditions during spring migration play an important role in breeding propensity and success (Cunningham et al., 2023; Schindler et al., 2024). Newly emergent grass leaves are more profitable to geese as they have higher protein content and lower fibre than older leaves (Fox, 1993; Hassall et al., 2001). By timing migration to follow emerging vegetation, geese may optimise nutrient gains and arrive at breeding areas in optimal condition with higher probability of nesting success. Although Greenland white‐fronted geese have advanced their departure from wintering areas, potentially in response to earlier springs in a warming climate (Fox et al., 2014), a mismatch may still occur if geese are not advancing migration at a rate commensurate with climate change (Visser & Both, 2005), contributing towards declines of fecundity and abundance. Greenland white‐fronted geese migrate from wintering to staging areas over the Atlantic Ocean and therefore make departure decisions based on environmental cues (e.g. temperature and timing of peak vegetation growth during spring) from wintering areas, rather than gradually following the flush of plant growth like herbivore species which migrate over land (Merkle et al., 2016; van Wijk et al., 2012). Warming is more pronounced at higher latitudes than in temperate regions (Francis et al., 2017; Rantanen et al., 2022). Consequently, these environmental cues on wintering areas may not be indicative of optimal staging or breeding conditions if spring phenology advances at different rates along migration routes, as seen in other Arctic‐nesting geese (Reséndiz‐Infante & Gauthier, 2024).

We found that earlier spring phenology on staging areas adversely affected fecundity of Islay, Loch Ken and Elsewhere, but not Wexford. These differences likely stem from differences in staging habitats; Wexford geese typically stage in western Iceland, while Islay and Loch Ken geese typically stage in southern Iceland, and Elsewhere geese in both areas (Fox et al., 1999, figure S5). GDD on staging areas used by Wexford geese was generally lower than those used by other subpopulations, potentially reducing phenological mismatches in goose arrival to staging areas and peak nutrients in plant foods. Habitat also varies among staging areas; geese predominantly use cultivated grasslands across staging areas but also use potato and barley fields in southern Iceland, which are uncommon habitats in western Iceland (Fox, 2003). Earlier springs may advance crop growing seasons, thus increasing disturbance of feeding geese by agricultural machinery just before the birds depart for breeding areas. GDD on wintering areas negatively affected fecundity of Loch Ken geese, suggesting they may not depart wintering areas early enough to optimise forage quality (i.e. depart after prolonged growing periods reduce newly emergent grass leaves). GDD on wintering areas positively affected fecundity of the Wexford subpopulation. The Wexford and Islay wintering areas both comprise land managed specifically for Greenland white‐fronted geese through local agreements with farmers and additional site protection designations (Fox, 2003), which all likely improve habitat quality relative to that experienced by other subpopulations. However, more research is needed to better understand how habitat management affects subsequent reproduction. We found no evidence that breeding area GDD affected fecundity in any of the four subpopulation groups. While late spring temperatures are warming in breeding areas in west Greenland, timing of snow melt and plant growth have not advanced relative to arrival of Greenland white‐fronted geese (i.e. breeding area GDD has not increased over our study period). Thus, warming temperatures during the breeding season may not benefit goose fecundity unless these changes correspond with earlier spring phenology, leading to improved foraging conditions upon breeding area arrival. Due to the spatial and temporal resolutions of our analyses, we used growing degree days as a proxy for spring vegetation phenology and assumed maximum forage nutrition coincided with peak spring plant growth, as demonstrated in other studies (Merkle et al., 2016; van der Graaf et al., 2006; van Wijk et al., 2012). Future research linking direct assessments of nutrient content of forage intake to reproductive outcomes in individually tracked geese could help us better understand these processes.

Cumulative snow on breeding areas negatively affected fecundity among the Elsewhere subpopulations. Boyd and Fox (2008) previously found a negative relationship between spring precipitation and percent young in Wexford and Islay subpopulations. A warming climate has increased spring snowfall on breeding areas, measured by the North Atlantic Oscillation index (Hoerling et al., 2001; Johannessen et al., 2004). This reduces feeding opportunities for geese upon arrival, hindering their replenishment of depleted energy stores after migration prior to breeding (Weegman et al., 2017). As the climate in Greenland continues to warm, increased spring precipitation may fall as rain (not snow), potentially advancing the breeding period and improving habitat quality (Boyd & Fox, 2008; Weegman et al., 2017), as with Svalbard‐breeding pink‐footed geese (Jensen et al., 2008, 2014). However, warmer temperatures and earlier breeding may create a mismatch in timing of brood rearing and optimal gosling food availability, reducing gosling survival, as observed in greater snow geese *Anser caerulescens atlantica* (Dickey et al., 2008) and barnacle geese *Branta leucopsis* (Lameris et al., 2018).

Shooting in Ireland and Great Britain was a major source of mortality before winter hunting bans were introduced from 1982, reflected in population growth from 1982 to 1999 (Fox, 2003) and the increased survival of Wexford (juvenile and adult) and Elsewhere (adult) birds following hunting protection in Iceland. Despite protections, illegal harvest of Greenland white‐fronted geese still occurs, especially during autumn staging in Iceland (Environment and Food Agency of Iceland, 2024). Decreased survival of Islay, Loch Ken, and Elsewhere geese following recent protections may be due to differential hunting pressure across staging areas; anecdotal evidence from neck collar recoveries and GPS tracking devices used in this study suggests hunting pressure may be strongest in southern Iceland where Islay, Loch Ken and some Elsewhere geese typically stage. However, survival estimates for Islay and Loch Ken during the early years of our study had large uncertainty due to sample size limitations, potentially limiting inference for these effects. We recommend future research focus on quantifying the role of illegal harvest on regulating Greenland white‐fronted goose population trends.

Severe storms during migration are a major source of mortality in migratory birds (Newton, 2024), especially birds such as Greenland white‐fronted geese that cross the North Atlantic and Greenland ice cap. Mortality of Arctic‐nesting geese and other migratory bird species during migration is markedly higher in juveniles than adults because juveniles are less efficient fliers and navigators (Menu et al., 2005; Newton, 2024; Owen & Black, 1989). However, storms may benefit geese during migration if providing strong tailwinds, which decrease energy use during flight. Thus, some adult geese may have increased survival by taking advantage of these opportunities.

Testing hypotheses about the effects of changing environmental conditions on animal survival, fecundity, and ultimately, population growth are fundamental objectives across animal ecology. With increased habitat fragmentation from anthropogenic land‐use change, accounting for demographic heterogeneity within animal populations will become increasingly important. Previous studies show population dynamics and responses to environmental changes vary among fragmented populations of many animal taxa across a gradient of life histories, including mammals (Bond et al., 2021), amphibians (Cayuela et al., 2020) and birds (Alisauskas et al., 2022; Millsap, 2018; Rushing et al., 2016; Schaub et al., 2015), confirming the need to account for demographic heterogeneity among animal populations to fully understand observed changes in abundance. Our metapopulation approach estimates subpopulation‐specific demographic rates, quantifies the contributions of these demographic rates to subpopulation growth, and tests hypothesised environmental drivers of subpopulation dynamics using a single hierarchical model. Use of IMMs is limited to systems in which demographic data are collected from multiple subpopulations, which may be challenging in some cases. However, IPMs (and by extension, IMMs) are powerful tools that can integrate data with variable sampling protocols and periods across multiple sites (Schaub et al., 2015; Zipkin & Saunders, 2018), providing opportunities for scientific collaboration across agencies and political borders towards common conservation goals. By accounting for subpopulation‐metapopulation dynamics in population monitoring efforts, we can better understand ecological responses to changing environmental conditions, allowing us to better address global challenges of conservation at large spatial scales in the face of pressing climate and land use changes.

## AUTHOR CONTRIBUTIONS

Alexander R. Schindler, Anthony D. Fox, Seán B. A. Kelly and Mitch D. Weegman conceived the ideas; Alexander R. Schindler, Anthony D. Fox, and Mitch D. Weegman designed methodology; Anthony D. Fox, Alyn J. Walsh and Larry R. Griffin collected and curated the data; Alexander R. Schindler analysed the data and led the writing of the manuscript. All authors contributed critically to the drafts and gave final approval for publication.

## CONFLICT OF INTEREST STATEMENT

The authors declare no conflicts of interest.

## STATEMENT OF INCLUSION

Our study brings together ecologists and conservation stakeholders from several different countries, including scientists based in the countries where the study was carried out. All authors were engaged early on with the research and study design to ensure that the diverse sets of perspectives they represent were considered from the onset of the study.

## Supporting information


**Appendix S1.** Additional methodological details, supplementary tables and figures related to the integrated metapopulation model to quantify environmental drivers of Greenland white‐fronted goose metapopulation dynamics.

## Data Availability

All data and code are available from the Dryad Digital Repository https://doi.org/10.5061/dryad.sqv9s4nd7 (Schindler et al., 2026).
