# Supplementary material for: Environmental drivers of metapopulation dynamics throughout the full annual cycle in a declining Arctic‐nesting migratory herbivore
Source: J Anim Ecol. 2026 Mar 5;95(5):865–81. doi: 10.1111/1365-2656.70236 (PMC13145331; doi:10.1111/1365-2656.70236)
Supplement: Supplementary file 1 — Appendix S1. Additional methodological details, supplementary tables and figures related to the integrated metapopulation model to quantify environmental drivers of Greenland white‐fronted goose metapopulation dynamics. [file JANE-95-865-s001.pdf]

Supporting Information for: Environmental drivers of metapopulation dynamics throughout the full annual cycle in a declining Arctic-nesting migratory herbivore

Alexander R. Schindler, Anthony D. Fox, Alyn J. Walsh, Larry R. Griffin, Seán B. A. Kelly and Mitch D. Weegman

Interpolating missing counts

The Elsewhere counts represented a sum of individual subpopulation counts within the group. A large proportion (90%) of study years contained missing data for at least one of these smaller subpopulations. Because our model could not estimate individual subpopulation sizes for subpopulations in the Elsewhere group, we could not account for missing count data for some of these subpopulations and include count data for others (i.e., would have to omit the entire Elsewhere count if even one of these subpopulations had missing data). We therefore used linear interpolation to fill missing data for individual subpopulation counts prior to summation.

We interpolated count data for one year at Wexford and between one and 18 total years for the 39 subpopulations with missing count data within the Elsewhere group (Table S1). The sum of Elsewhere counts without interpolation equalled 97% of the sum of Elsewhere counts with interpolation (annual counts without interpolation were 87%-100% of annual counts with interpolation). Given the relative completeness of the count data and Greenland white-fronted goose life history characteristics (i.e., high annual survival and low fecundity [Weegman et al. 2016, 2022], which limit the potential for large annual changes in population size), interpolation of count data had minimal capacity to affect estimates of population size but was necessary to make count data comparable among years.

Delineating timing and spatial areas used by subpopulations throughout the annual cycle

To determine space use throughout the annual cycle, we used data collected from 57 GPS tracking devices deployed from 2012 to 2022 on captured Greenland white-fronted geese from our focal subpopulations (Ozsanlav-Harris 2023, Schindler et al. 2024). We calculated subpopulation-specific minimum convex polygons using 90% of GPS points to represent core space use for breeding, staging, migration and wintering phases (Figures S1 – S5). We compared these distributions to historical data, including breeding season aerial survey data (Malecki et al. 2000), staging area collar resighting data (Fox et al. 1983, Francis & Fox 1987) and wintering census data (Fox et al. 1998), and confirmed no shifts in breeding, staging, or wintering distributions during our time series. While we could not compare GPS information about migration flights to historical data, these are direct, continuous flights across the ocean and Greenland ice caps. Since breeding, staging, and wintering distributions have not changed, the flight paths over the ocean are unlikely to have changed either. We combined GPS information from all subpopulations to create 90% minimum convex polygons for Elsewhere migration, and used the census, aerial survey and collar resighting data to define Elsewhere wintering, breeding and staging areas (Figures S1 – S5). We further subset breeding space use to areas below 200 m

elevation above sea level, as these are important feeding areas for Greenland white-fronted geese during the breeding season (Fox et al. 1983; Figure S1).

Observers recorded time of goose departure from wintering areas at each of our focal subpopulations. Goose departure from wintering areas has advanced over time (Fox and Walsh 2012); we fit a generalised linear model with a Poisson distribution to quantify change in departure date over time, predict missing departure dates for focal subpopulations and define departure dates for the Elsewhere category. We defined arrival on staging areas as one day post-departure from wintering areas based on migration duration information from GPS tracking devices. Goose departure dates from spring staging areas have not changed over time; we set a fixed date of 7 May for arrival on breeding areas based on observed departures from staging areas and GPS tracking information (Fox and Walsh 2012, Fox et al. 2014). We did not observe any trends in departure from breeding areas or autumn staging areas in GPS tracking data from 2012 to 2022. We defined breeding and autumn staging departure days from means among all geese and years with GPS tracking data.

Calculating weather metrics

We calculated cumulative growing degree days (GDD) from 1 January through the day of departure for spring migration on wintering areas, through the day of arrival on spring staging areas, and through the day of arrival on breeding areas (Figure S6). We defined the temperature threshold for plant growth (T_{BASE}) as -5° C for our study areas (Botta et al., 2000). We calculated growing degree units (GDU) for day i as $GDU^i = T^i_{AVG} - T_{BASE}$ when $T^i_{AVG} > T_{BASE}$ and 0 otherwise (i.e., no growth occurred when the average daily temperature, T^i_{AVG}, was ≤ -5° C; van Wijk et al., 2012). The GDD of the corresponding day of departure/arrival, k, was the accumulation of growing degree units from 1 January to the corresponding day of interest (van Wijk et al., 2012):

$$GDD^k = \sum_{i=1}^{k} GDU^i$$

We calculated cumulative snowfall on breeding areas from the start of winter (defined as the day when the daily maximum temperature for >50% of the corresponding breeding area remained below 0° C until spring) until geese arrived (Figure S6). We defined snowfall as precipitation when the maximum daily temperature was below 0° C, and cumulative snowfall as the cumulative sum of average daily snowfall within the breeding area. Severe storms are characterized by sudden drops in barometric pressure (Saucier, 2003). Using average daily barometric pressure and average daily differences in barometric pressure in migration areas, we identified the cumulative number of severe storms occurring within 7 days before or after departures from wintering, spring staging, breeding, and autumn staging areas, using the severe storm metric from Schindler et al. (2024; Figure S6).

Multistate capture-recapture model

We estimated survival (φ), movement (ψ) and resighting (p) probabilities using a multistate capture-recapture model with a multinomial likelihood. The multistate capture-recapture data contained nine states (1: juvenile (J) at Wexford (W), 2: juvenile at Islay (Is), 3: juvenile at Loch Ken (LK), 4: juvenile Elsewhere (E), 5: adult (A) at Wexford, 6: adult at Islay, 7: adult at Loch Ken, 8: adult Elsewhere, 9: dead). There were no resightings in states 1–4 as surviving geese marked as juveniles returned to wintering areas as adults in the subsequent year. We did not have enough juvenile geese captured Elsewhere (i.e., observations for state 4) to include in our analysis; thus, we defined probabilities of juveniles Elsewhere returning as adults in any subpopulation or dying as 0 and did not estimate them. We defined the state-transition matrix $\mathbf{\Psi}$ as a 9×9 matrix for transition probabilities of an individual in a given state at time t (row) to a state at time $t + 1$ (column):

$$
\boldsymbol{\Psi}_t = \begin{bmatrix}
0 & 0 & 0 & 0 & \varphi^J_{W,t}(1-\psi^J_{W,Is,t} - \psi^J_{W,LK,t} - \psi^J_{W,E,t}) & \varphi^J_{W,t}\psi^J_{W,Is,t} & \varphi^J_{W,t}\psi^J_{W,LK,t} & \varphi^J_{W,t}\psi^J_{W,E,t} & 1-\varphi^J_{W,t} \\
0 & 0 & 0 & 0 & \varphi^J_{Is,t}\psi^J_{Is,W,t} & \varphi^J_{Is,t}(1-\psi^J_{Is,W,t} - \psi^J_{Is,LK,t} - \psi^J_{Is,E,t}) & \varphi^J_{Is,t} \times \psi^J_{Is,LK,t} & \varphi^J_{Is,t} \times \psi^J_{Is,E,t} & 1-\varphi^J_{Is,t} \\
0 & 0 & 0 & 0 & \varphi^J_{LK,t}\psi^J_{LK,W,t} & \varphi^J_{LK,t}\psi^J_{LK,Is,t} & \varphi^J_{LK,t}(1-\psi^J_{LK,W,t} - \psi^J_{LK,Is,t} - \psi^J_{LK,E,t}) & \varphi^J_{LK,t} \times \psi^J_{LK,E,t} & 1-\varphi^J_{LK,t} \\
0 & 0 & 0 & 0 & 0 & 0 & 0 & 0 & 0 \\
0 & 0 & 0 & 0 & \varphi^A_{W,t}(1-\psi^A_{W,Is,t} - \psi^A_{W,LK,t} - \psi^A_{W,E,t}) & \varphi^A_{W,t}\psi^A_{W,Is,t} & \varphi^A_{W,t}\psi^A_{W,LK,t} & \varphi^A_{W,t}\psi^A_{W,E,t} & 1-\varphi^A_{W,t} \\
0 & 0 & 0 & 0 & \varphi^A_{Is,t}\psi^A_{Is,W,t} & \varphi^A_{Is,t}(1-\psi^A_{Is,W,t} - \psi^A_{Is,LK,t} - \psi^A_{Is,E,t}) & \varphi^A_{Is,t}\psi^A_{Is,LK,t} & \varphi^A_{Is,t}\psi^A_{Is,E,t} & 1-\varphi^A_{Is,t} \\
0 & 0 & 0 & 0 & \varphi^A_{LK,t}\psi^A_{W,E,t} & \varphi^A_{LK,t}\psi^A_{LK,Is,t} & \varphi^A_{LK,t}(1-\psi^A_{LK,W,t} - \psi^A_{LK,Is,t} - \psi^A_{LK,E,t} & \varphi^A_{LK,t}\psi^A_{LK,E,t} & 1-\varphi^A_{LK,t} \\
0 & 0 & 0 & 0 & \varphi^A_{E,t}\psi^A_{E,W,t} & \varphi^A_{E,t}\psi^A_{E,Is,t} & \varphi^A_{E,t}\psi^A_{E,LK,t} & \varphi^A_{E,t}(1-\psi^A_{E,W,t} - \psi^A_{E,Is,t} - \psi^A_{E,LK,t} & 1-\varphi^A_{E,t} \\
0 & 0 & 0 & 0 & 0 & 0 & 0 & 0 & 1
\end{bmatrix}
$$

We defined the vector of state-specific resighting probabilities as:

$$\boldsymbol{p}_t = [0 \quad 0 \quad 0 \quad 0 \quad p_{W,t} \quad p_{Is,t} \quad p_{LK,t} \quad p_{E,t} \quad 0]$$

where p_W, p_{Is}, p_{LK} and p_E were the resighting probabilities at Wexford, Islay, Loch Ken and Elsewhere, respectively.

Goodness-of-fit tests

We examined goodness-of-fit of our integrated metapopulation model (IMM) using posterior predictive checks applied to the fecundity, survival and dispersal and population sub-models (Schaub and Kéry 2021, Frost et al. 2023, Christian et al. 2023). These involved randomly sampling demographic and movement rate estimates from our IMM posteriors and using these estimates to simulate replicate datasets of each data type used in our model. We then compared real and simulated datasets graphically and computed Bayesian p-values using Freeman-Tukey discrepancy measures.

Fecundity and population sub-models

Let $\boldsymbol{\theta}$ denote a vector of all estimated demographic rates, movement rates, and observation variation in count data in the IMM. We randomly selected 1000 posterior samples $\boldsymbol{\theta}_s, s = 1, \dots, 1000$ from the 78000 posterior samples in our IMM. We simulated age- and subpopulation-specific abundance under our fecundity and population sub-models (specified in the main manuscript) using the values of demographic rates, movement rates, and initial sizes of each age class in each subpopulation for each of these posterior draws. Then we drew juvenile count data ($J_{i,t,s}^{obs}$ for subpopulation i in year t for sample s) each year according to our juvenile count model:

$$J_{i,t,s}^{obs} \sim Poisson(J_{i,t,s})$$

and count data in each year according to our count model:

$$y_{i,t,s} \sim logNormal(\log(Ntot_{i,t,s}), \tau_s^y).$$

We obtained the expected number of juvenile and total counts by calculating expected age sizes for each subpopulation in all years from the fecundity and population sub-models. We compared the real and simulated data sets graphically (Figures S7, S9).

For a quantitative assessment of fit, we used the Freeman-Tukey discrepancy measure to compare expected and observed counts of each type (juvenile and total counts per subpopulation and year) over the whole study period. For two vectors $\boldsymbol{u}, \boldsymbol{v}$ of length T (i.e., 39 years in the study), the Freeman-Tukey discrepancy was defined as:

$$FT(\boldsymbol{u}, \boldsymbol{v}) = \sum_{t=1}^{T} \left(\sqrt{u_t} - \sqrt{v_t}\right)^2.$$

For each posterior sample s, we calculated the Freeman-Tukey discrepancy between the expected and observed counts for the replicate and real data:

$$FT(y_s^{rep}, \mathbf{E}y_s), FT(y^{real}, \mathbf{E}y_s).$$

We defined the Bayesian p-value as the proportion of replicates for which the discrepancy between observed and expected counts in the replicate data exceeds that of the real data (Figures S8, S10):

$$p_B = \frac{1}{1000}\sum_{s=1}^{1000} I\left(FT\left(y_s^{rep}, \mathbf{E}y_s\right) > \ FT\left(y^{real}, \mathbf{E}y_s\right)\right).$$

Survival and dispersal sub-model

We randomly sampled 1000 draws of all estimated demographic rates, movement rates, and resighting probabilities from our IMM posteriors. We generated 1000 replicate capture-mark-resighting datasets $\boldsymbol{CMR}_s, s = 1, \dots, 1000$ by simulating states for each captured individual for each posterior draw beginning the year following initial capture using our state-transition matrices $\boldsymbol{\Psi}_{t,s}$, then simulated resightings using our resighting probability vectors $\boldsymbol{p}_{t,s}$. Each replicate dataset had the same structure as the real data, i.e., the same number of captures per state and year. We then summarised both simulated and real capture histories by calculating the total number of resightings per state for each year t for each posterior sample s ($\boldsymbol{D}_{t,s}$), as well as the total number of resightings among all states per year for each posterior sample. We compared the real and simulated datasets graphically (Figs. S11, S12).

We calculated the expected number of resightings per subpopulation and year as the sum of the expected number of resightings per cohort (i.e., individuals captured in the same year). The expected number of resightings among all cohorts C in year t per state for each posterior sample s ($\mathbf{E}\boldsymbol{D}_{t,s}$) was defined as:

$$\mathbf{E}\boldsymbol{D}_{t,s} = \sum_{c=1}^{C} \boldsymbol{f}_c \left(\prod_{\delta=c}^{t-1} \boldsymbol{\Psi}_{\delta,s}\right) \boldsymbol{p}_{t,s}$$

where $\boldsymbol{f}_c$ was a vector containing the number of individuals from cohort c captured in each state during the year of capture, $\boldsymbol{\Psi}_{\delta,s}$ were the state-transition matrices (defined above) for years $\delta = c, \dots, t-1$, the elementwise product of transition matrices were $\prod_{\delta=c}^{t-1} \boldsymbol{\Psi}_{\delta,s} = \boldsymbol{\Psi}_{c,s} \circ \dots \circ \boldsymbol{\Psi}_{t-1,s}$, and $\boldsymbol{p}_t$ were the vectors of resighting probabilities (defined above). The total expected number of resightings was the sum of the expected resightings among all cohorts and states. For a quantitative assessment of fit, we used the Freeman-Tukey discrepancy measure to compare expected and observed number of resightings over the whole study period in an identical manner to the count data (Figures S11, S13).

Population growth rate expressions for tLTRE analyses

Our IMM estimated number of total immigrants per subpopulation and year ($I_{i,t}$). We calculated post hoc age- and subpopulation-specific immigration rates (ω) for each subpopulation and year. For example, we used the posterior distributions for number of juveniles, number of adults, survival and movement probabilities to calculate immigration rates at Wexford as:

$$\omega^{J}_{Is,W,t} = \varphi^{J}_{Is,t-1}\psi^{J}_{Is,W,t-1}J_{Is,t-1}/Ntot_{W,t-1}$$
$$\omega^{A}_{Is,W,t} = \varphi^{A}_{Is,t-1}\psi^{A}_{Is,W,t-1}A_{Is,t-1}/Ntot_{W,t-1}$$
$$\omega^{J}_{LK,W,t} = \varphi^{J}_{LK,t-1}\psi^{J}_{LK,W,t-1}J_{LK,t-1}/Ntot_{W,t-1}$$
$$\omega^{A}_{LK,W,t} = \varphi^{A}_{LK,t-1}\psi^{A}_{LK,W,t-1}A_{LK,t-1}/Ntot_{W,t-1}$$
$$\omega^{A}_{E,W,t} = \varphi^{A}_{E,t-1}\psi^{A}_{E,W,t-1}A_{E,t-1}/Ntot_{W,t-1}$$

We then defined realised population growth rates based on age- and subpopulation-specific survival, fecundity and immigration rates and proportional abundance of each stage class (pJ, p$N1$, pNad and pI, constrained to sum to 1). For example, we defined realised population growth rates of Wexford defined as:

$$\begin{aligned}\lambda_t = \Big(&\big(\mathrm{p}N1_{W,t} + \mathrm{p}Nad_{W,t} + \mathrm{p}I_{W,t}\big)\varphi^{A}_{W,t}\psi^{A}_{W,W,t}\gamma_{W,t} + \omega^{J}_{Is,W,t}\gamma_{W,t} + \omega^{J}_{LK,W,t}\gamma_{W,t} + \omega^{A}_{Is,W,t}\gamma_{W,t}\\ &+ \omega^{A}_{LK,W,t}\gamma_{W,t} + \omega^{A}_{E,W,t}\gamma_{W,t} + \mathrm{p}J_{W,t}\varphi^{J}_{W,t}\psi^{J}_{W,W,t} + \omega^{J}_{Is,W,t} + \omega^{J}_{LK,W,t}\\ &+ \big(\mathrm{p}N1_{W,t} + \mathrm{p}Nad_{W,t} + \mathrm{p}I_{W,t}\big)\varphi^{A}_{W,t}\psi^{A}_{W,W,t} + \omega^{A}_{Is,W,t} + \omega^{A}_{LK,W,t} + \omega^{A}_{E,W,t}\Big)\\ &/\big(\mathrm{p}J_{W,t} + \mathrm{p}N1_{W,t} + \mathrm{p}Nad_{W,t} + \mathrm{p}I_{W,t}\big)\end{aligned}$$

Calculating net immigration rates

To determine if subpopulations functioned as sources (i.e., emigration > immigration) or sinks (i.e., emigration < immigration), we calculated post hoc net immigration rates for adult geese for each subpopulation group. For example, we used the posterior distributions for number of adults, adult survival and adult movement probabilities to calculate net immigration rates at Wexford as:

$$E_{W,t} = \varphi^{A}_{W,t-1}\psi^{A}_{W,Is,t-1}A_{W,t-1} + \varphi^{A}_{W,t-1}\psi^{A}_{W,LK,t-1}A_{W,t-1} + \varphi^{A}_{W,t-1}\psi^{A}_{W,E,t-1}A_{W,t-1}$$
$$I_{W,t} = \varphi^{A}_{Is,t-1}\psi^{A}_{Is,W,t-1}A_{Is,t-1} + \varphi^{A}_{LK,t-1}\psi^{A}_{LK,W,t-1}A_{LK,t-1} + \varphi^{A}_{E,t-1}\psi^{A}_{E,W,t-1}A_{E,t-1}$$
$$I^{net}_{W,t} = \big(I_{W,t} - E_{W,t}\big)/A_{W,t-1}$$

where E were the number of emigrants, I the number of immigrants and I^{net} the net immigration rate, expressed as a proportion comprising emigration subtracted from immigration relative to the total number of adults in the subpopulation. Thus, the subpopulation functioned as a sink when $I^{net} > 0$ and a source when $I^{net} < 0$. We did not evaluate the contribution of juvenile movements to source-sink dynamics as we were unable to estimate juvenile survival or movement from Elsewhere.

References

Botta, A., Viovy, N., Ciais, P., Friedlingstein, P., & Monfray, P. (2000). A global prognostic scheme of leaf onset using satellite data. *Glob Change Biol*, 6, 709–725.

Christian, M., Oosthuizen, W. C., Bester, M. N., & de Bruyn, P. J. N. (2024). Robustly estimating the demographic contribution of immigration: Simulation, sensitivity analysis and seals. *J Anim Ecol*, 93, 632-645.

Francis, I. S, & Fox, A. D. (1987). Spring migration of Greenland white-fronted geese through Ireland. *Wildfowl*, 38, 7-12.

Fox, A. D., Madsen, J., & Stroud, D. A. (1983). A review of the summer ecology of the Greenland white-fronted goose *Anser albifrons flavirostris*. *Dansk Ornithol Foren Tidsskr*, 77, 43–55.

Fox, A. D., Norriss, D. W., Stroud, D. A., Wilson, H. J., & Merne, O. J. (1998). The Greenland white-fronted goose *Anser albifrons flavirostris* in Ireland and Britain 1982/83–1994/95: population change under conservation legislation. *Wildlife Biol*, 4, 1–12.

Fox, A. D., & Walsh, A. (2012). Warming winter effects, fat store accumulation and timing of spring departure of Greenland white-fronted geese *Anser albifrons flavirostris* from their winter quarters. *Hydrobiologia*, 697, 95–102.

Fox, A. D., Weegman, M. D., Bearhop, S., Hilton, G. M., Griffin, L., Stroud, D. A., *et al*. (2014). Climate change and contrasting plasticity in timing of a two-step migration episode of an Arctic-nesting avian herbivore. *Curr Zool*, 60, 233–242.

Frost, F., McCrea, R., King, R., Gimenez, O., & Zipkin, E. (2023). Integrated population models: achieving their potential. *J Stat Theory Pract*, 17, 6.

Malecki, R. A., Fox, A. D., & Batt, B. D. J. (2000). An aerial survey of nesting greater white-fronted and Canada geese in west Greenland. *Wildfowl*, 51, 49–58.

Ozsanlav-Harris, L. (2023). The Greenland white-fronted goose: diagnosing causes, identifying solutions. Ph.D. dissertation, University of Exeter, UK.

Saucier, W. J. (2003). Principles of meteorological analysis. Dover Publications, Mineola, USA.

Schaub, M., & Kéry, M. (2021). *Integrated population models: theory and ecological applications with R and JAGS.* Academic Press, London, UK. 622 pp.

Schindler, A. R., Fox, A. D., Wikle, C. K., Ballard, B. M., Walsh, A. J., Kelly, S. B. A., *et al*. (2024). Energetic trade-offs in migration decision-making, reproductive effort and subsequent parental care in a long-distance migratory bird. *P R Soc B*, 29, 20232016.

van Wijk, R. E., Kölzsch, A., Kruckenberg, H., Ebbinge, B. S., Müskens, G. J. D. M., & Nolet, B. A. (2012). Individually tracked geese follow peaks of temperature acceleration during spring migration. *Oikos*, 121, 655–664.

Weegman, M. D., Bearhop, S., Fox, A. D., Hilton, G. M., Walsh, A. J., McDonald, J. L., *et al*. (2016). Integrated population modelling reveals a perceived source to be a cryptic sink. *J Anim Ecol*, 85, 467–475.

Weegman, M. D., Walsh, A. J., Ogilvie, M. A., Bearhop, S., Hilton, G. M., Hodgson, D. J., & *et al*. (2022). Adult survival and per-capita production of young explain dynamics of a long-lived goose population. *Ibis*, 164, 574–580.

FIGURES AND TABLES

Table S1. Number of years with missing count data and mean count of smaller subpopulations included in the combined Elsewhere group in the integrated metapopulation model.

Subpopulation	Number of years with missing counts	Mean count
Inny Valley	22	0.72
Sullom Voe	33	3.14
Kilcolman	4	5.61
Bunduff	15	6.16
North Uist	14	6.42
Grindon	11	9.07
Tankerness, Orkney	12	15
Lower Lough Derg	14	15.12
Fergus and Shannon Estuaries	15	15.44
Benbecula	13	20.33
Assapol	6	20.74
Jura: Lowlandman's Bay	12	20.82
Blasket Islands	21	23.21
Chaluim Chille, Skye	0	24.73
Doo Lough	22	25.5
Loch Urrahag	0	28.42
Moine Mhor	0	30.27
Killarney Valley	13	30.85
Muck	16	30.88
Askernish	0	32.6
Stabannan	13	32.93
River Nore	9	33.68
Broadford, Skye	1	35.82
Tullagher	3	36.14
Fidden	1	37.67
Lough Oughter	7	38.09
North County Clare	6	42.53
Stronsay, Orkney	16	43.38
Connemara	20	44.7
Shiel	1	48.15
Caledon	11	49.59
North Lough Ree	13	55.33
Lower Lough Corrib	4	65.92
Drumharlow Lough	15	77.84
Lough Macnean	6	79.79
Loons, Orkney	0	86.88
Lough Conn	0	87.12

Dyfi	0	87.9
Rahasane Turlough	1	90.85
Bog of Erris	2	91.08
Errif and Derrycraff	5	94.83
Loch Bee	0	99.03
Lough Kilglass and Forbes	18	100.77
Pettigo	1	104.03
Rostaff and Killower	1	105.18
Sheskinmore Lough	1	111.85
Colonsay	0	121.85
Clachan	17	138.65
Dunfanaghy	2	142.92
Bute	0	169.68
Mey	0	170.95
Westfield	0	175.95
Endrich Mouth	0	223.05
Danna, Keills, Ulva	0	250.57
River Suck	0	254
Lough Gara	2	262.97
Benderloch, Lismore	0	266.52
Midland Lakes	2	307.97
Little Brosna	0	333.58
Stranraer	0	384.28
Coll	0	519.92
Loughs Foyle & Swilly	0	763.25
Tiree	0	924.18
Rhunahaorine	0	954.08
Machrihanish	0	1226.17

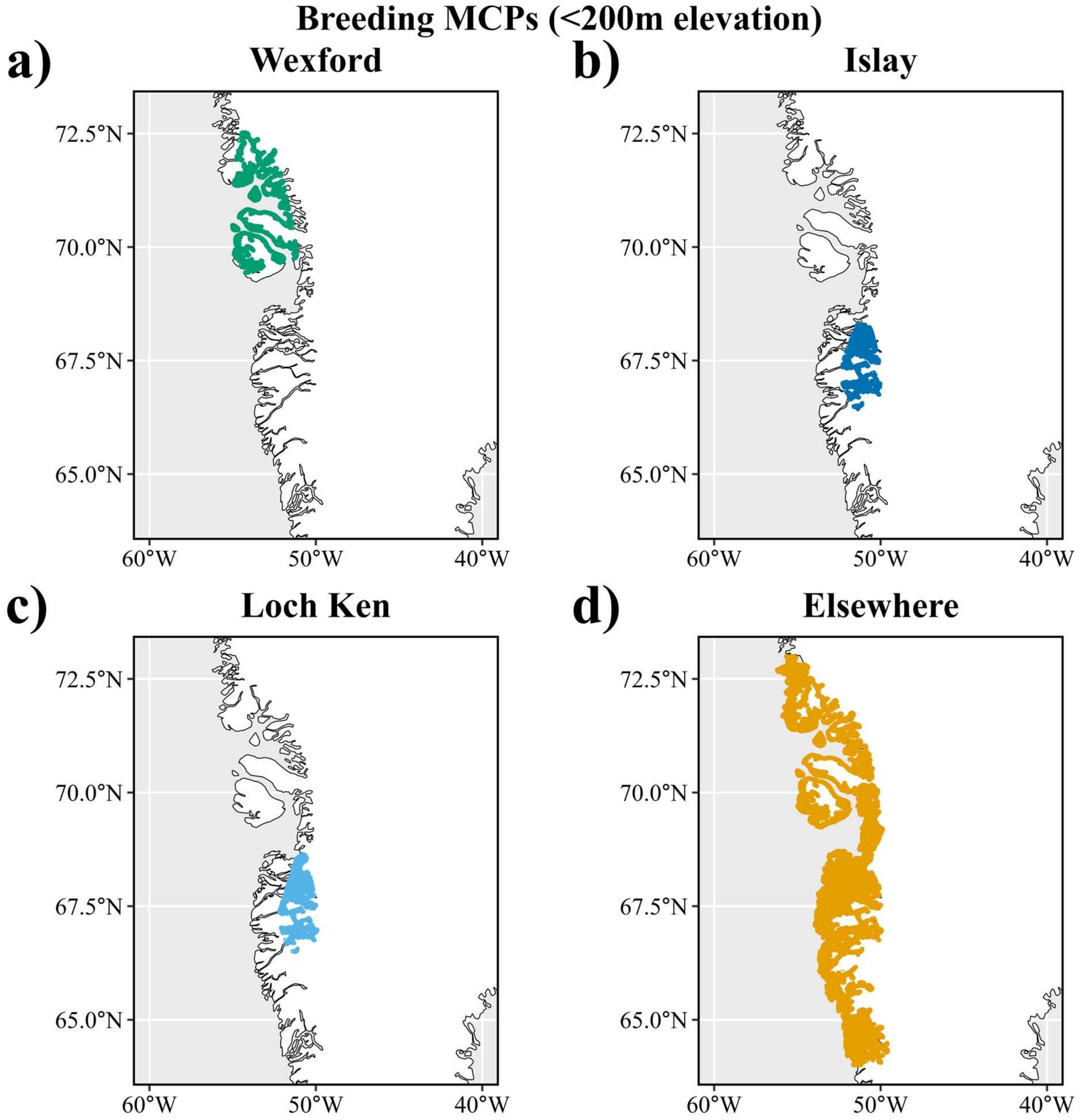

Figure S1. 90% minimum convex polygons (MCPs) representing the areas used by each focal subpopulation and Elsewhere during the breeding season. All breeding season MCPs were subset to areas <200 m above sea level, as these areas contain the breeding season feeding habitats.

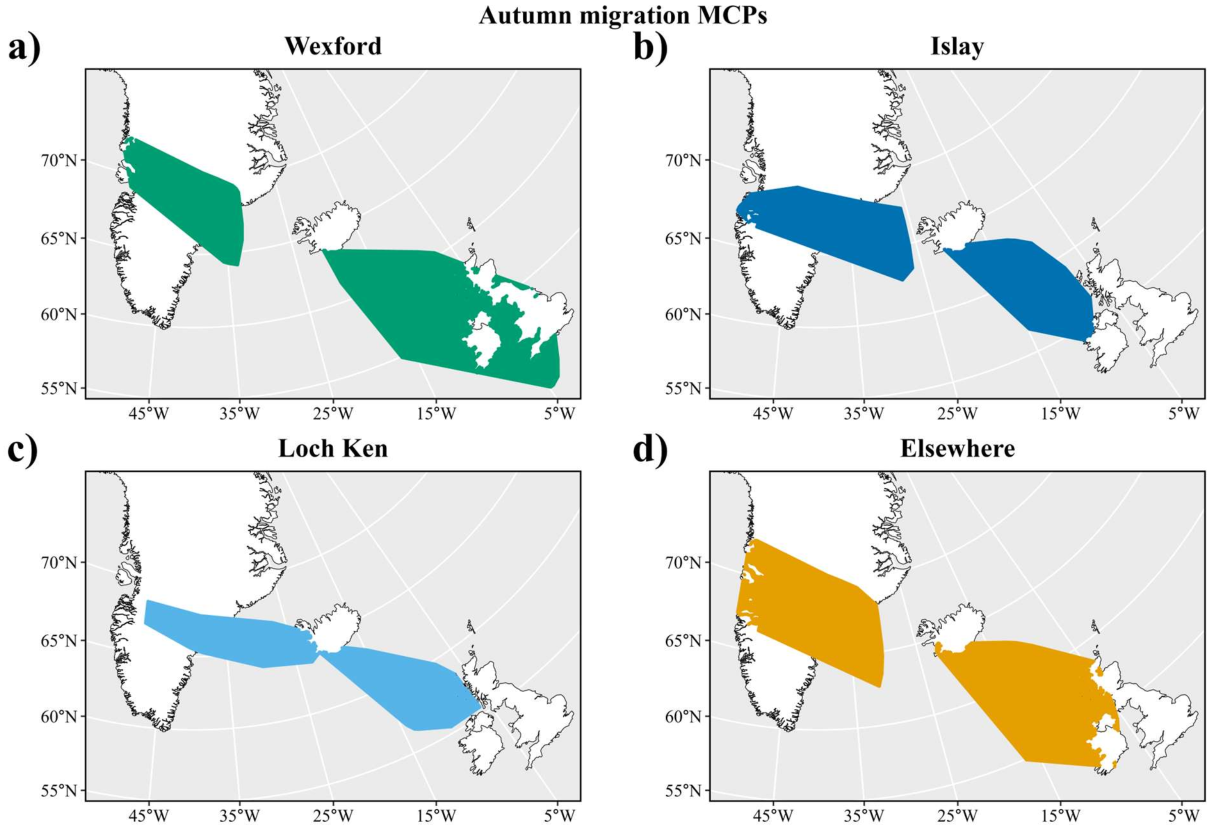

Figure S2. 90% minimum convex polygons (MCPs) representing the areas used by each focal subpopulation and Elsewhere during autumn migration.

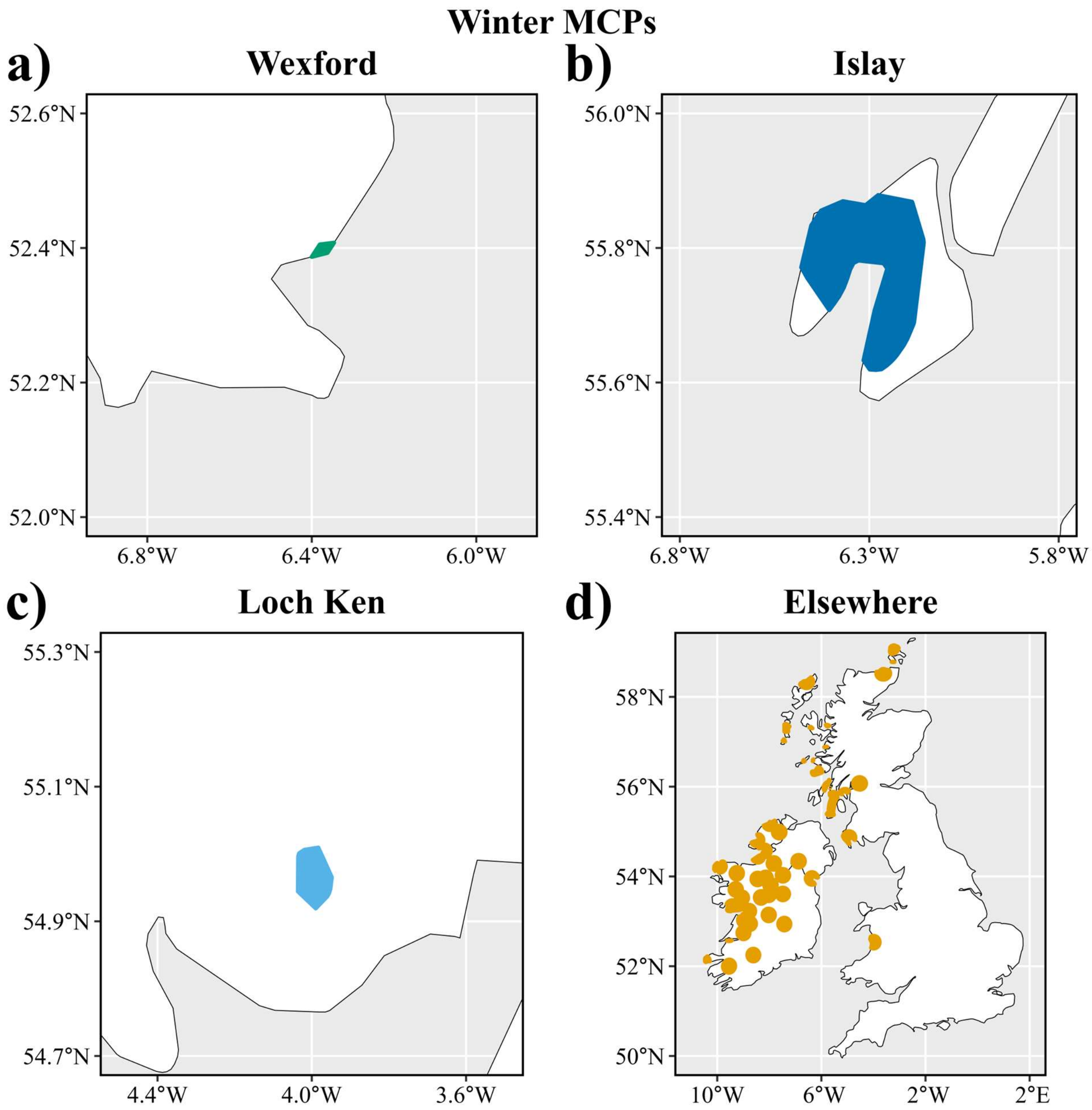

Figure S3. 90% minimum convex polygons (MCPs) representing the areas used by each focal subpopulation and Elsewhere during winter.

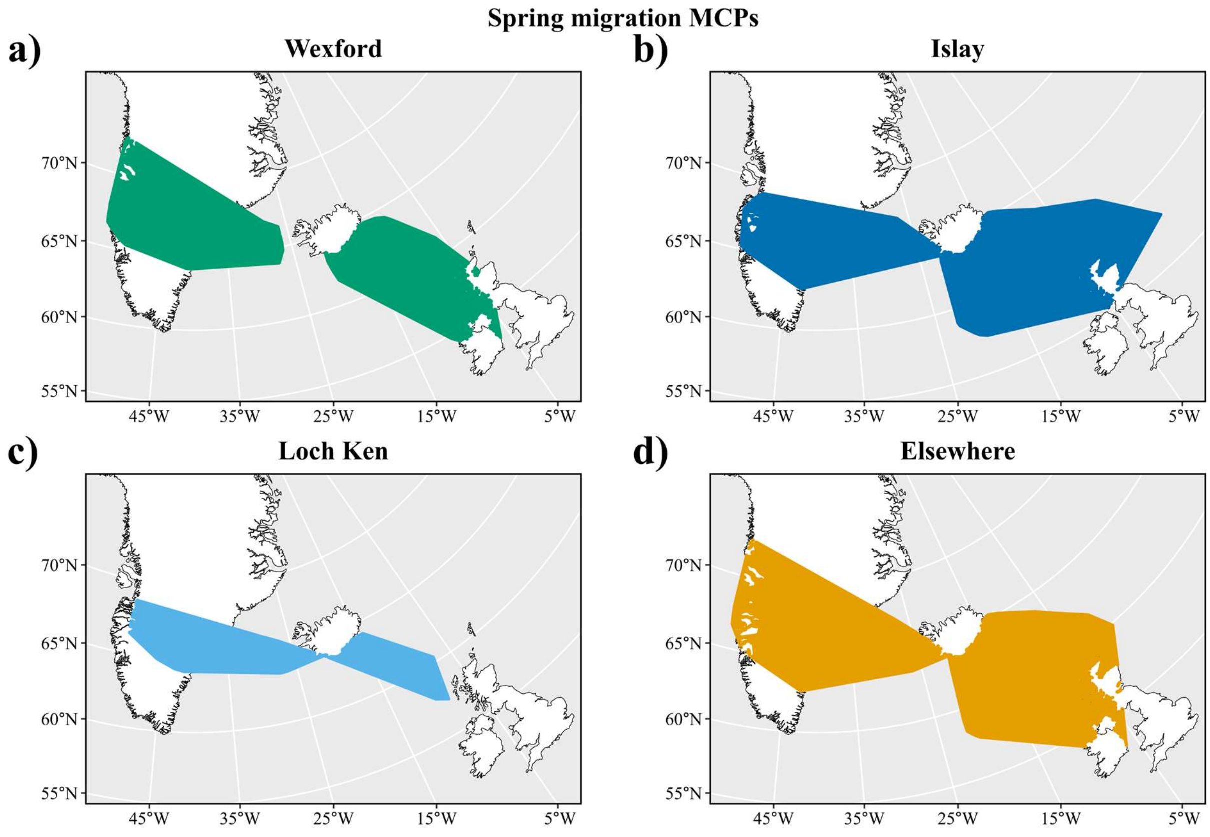

Figure S4. 90% minimum convex polygons (MCPs) representing the areas used by each focal subpopulation and Elsewhere during spring migration.

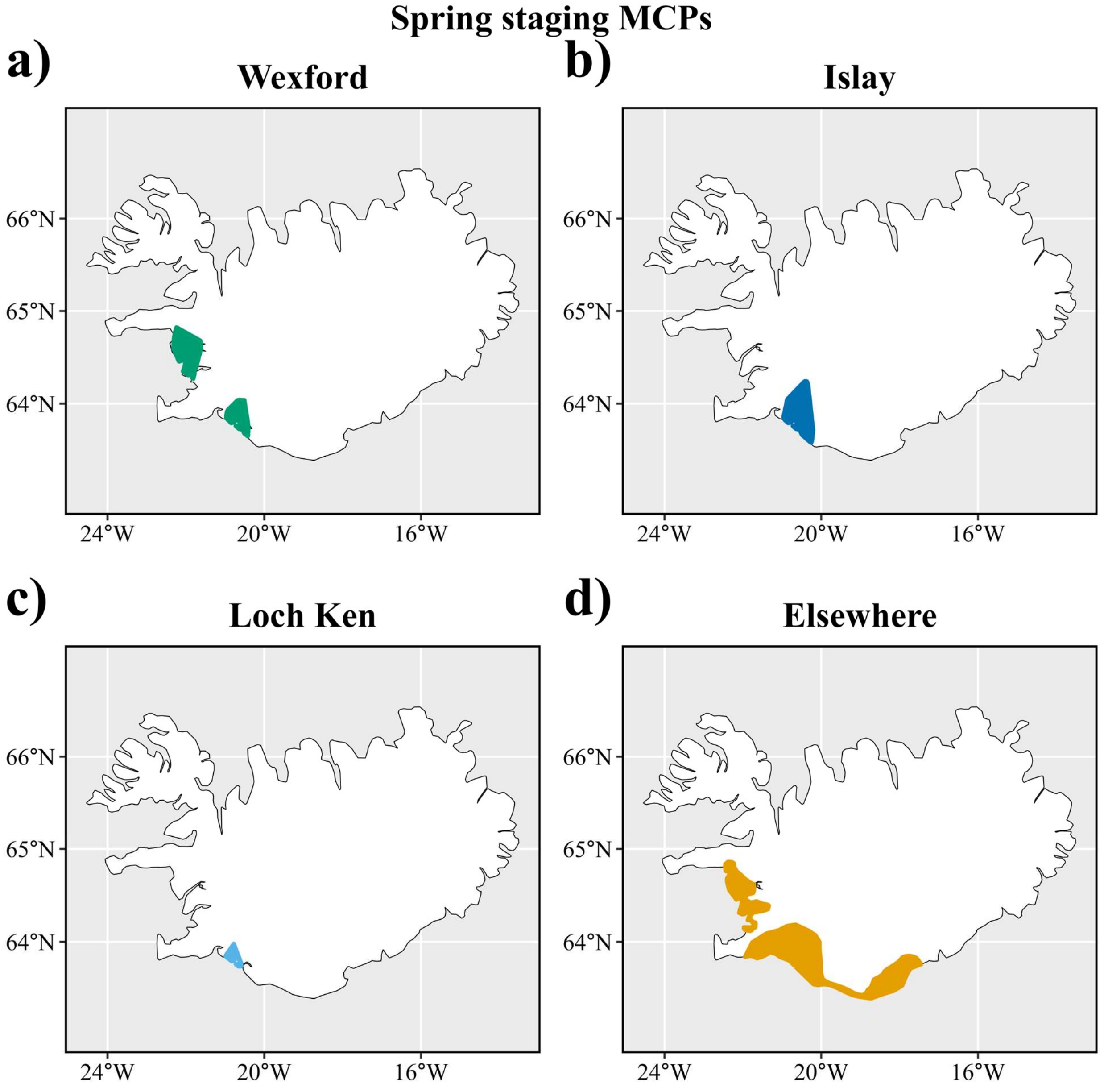

Figure S5. 90% minimum convex polygons (MCPs) representing the areas used by each focal subpopulation and Elsewhere during spring staging.

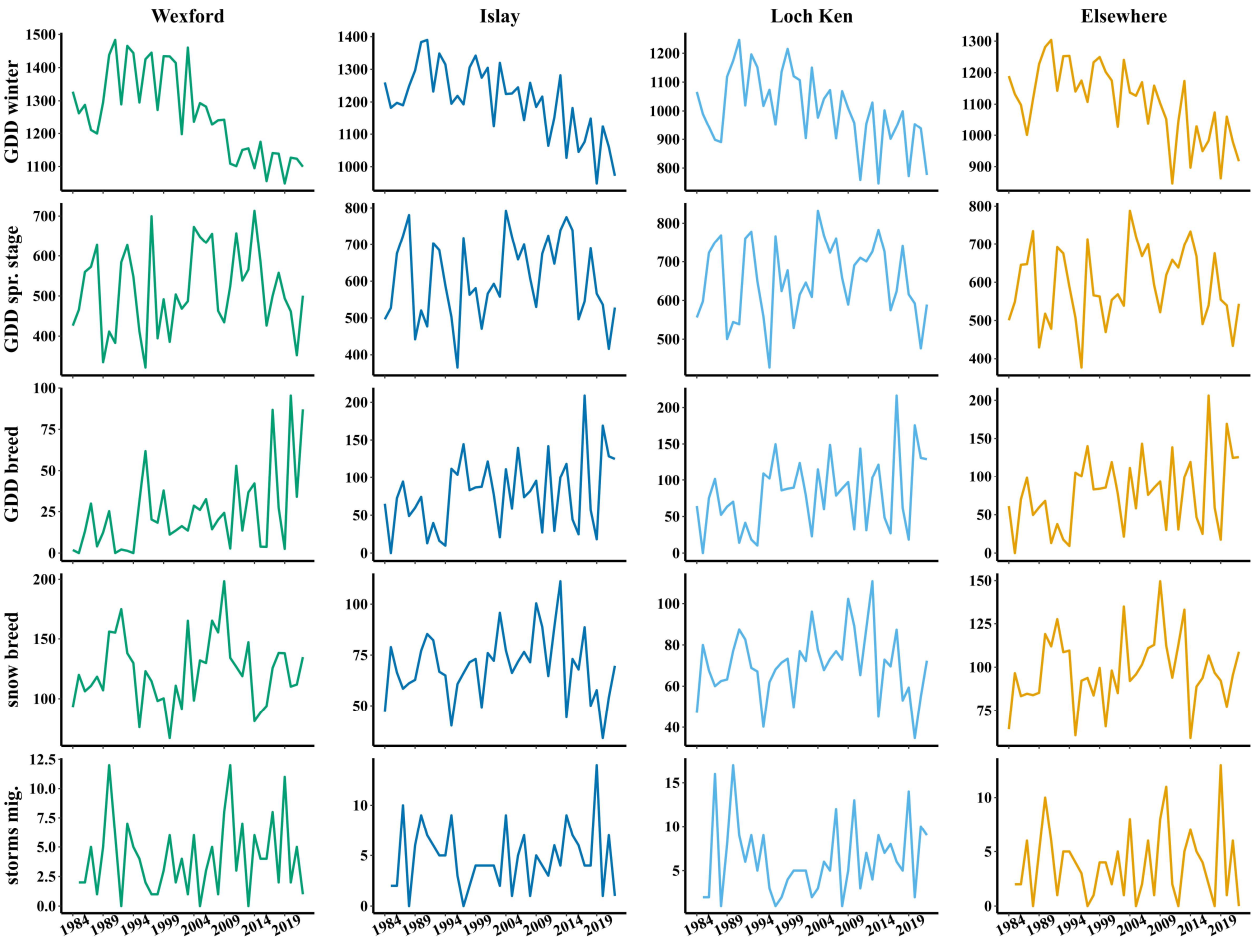

Figure S6. Annual values for all environmental variables used to test hypothesised environmental drivers of Greenland white fronted goose demography. Variables included cumulative growing degree days from 1 January through the day of departure for spring migration on wintering areas (GDD winter), through the day of arrival on spring staging areas (GDD spr. stage), and through the day of arrival on breeding areas (GDD breed), cumulative snowfall on breeding areas from the start of winter until geese arrived (snow breed) and the cumulative number of severe storms occurring within 7 days before or after departures from wintering, spring staging, breeding, and autumn staging areas (storms mig.).

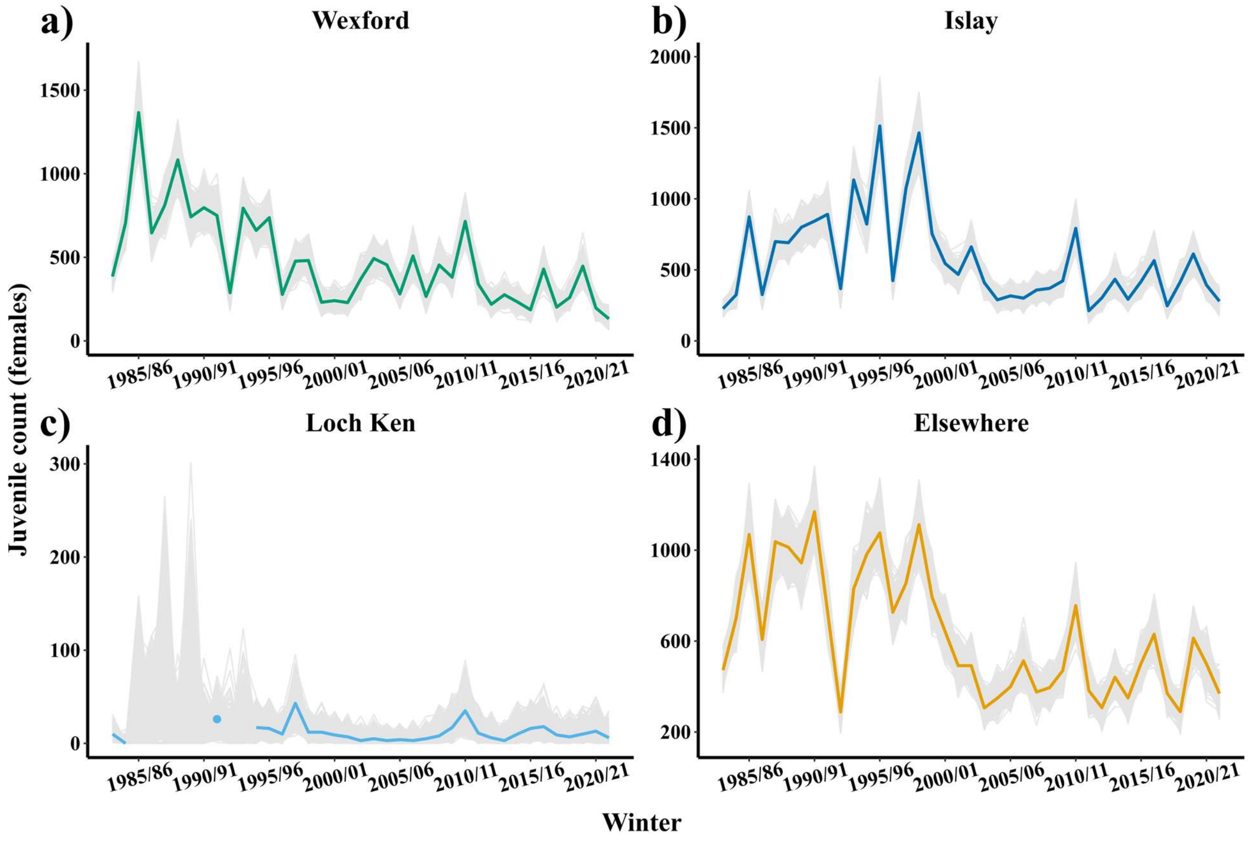

Figure S7. Data used in posterior predictive checks for the fecundity sub-model in the integrated metapopulation model. Coloured lines depict observed number of juveniles from the fecundity dataset and grey lines depict replicate juvenile count data generated from 1000 randomly selected posterior draws from estimated demographic rates, movement rates, and observation variation in count data.

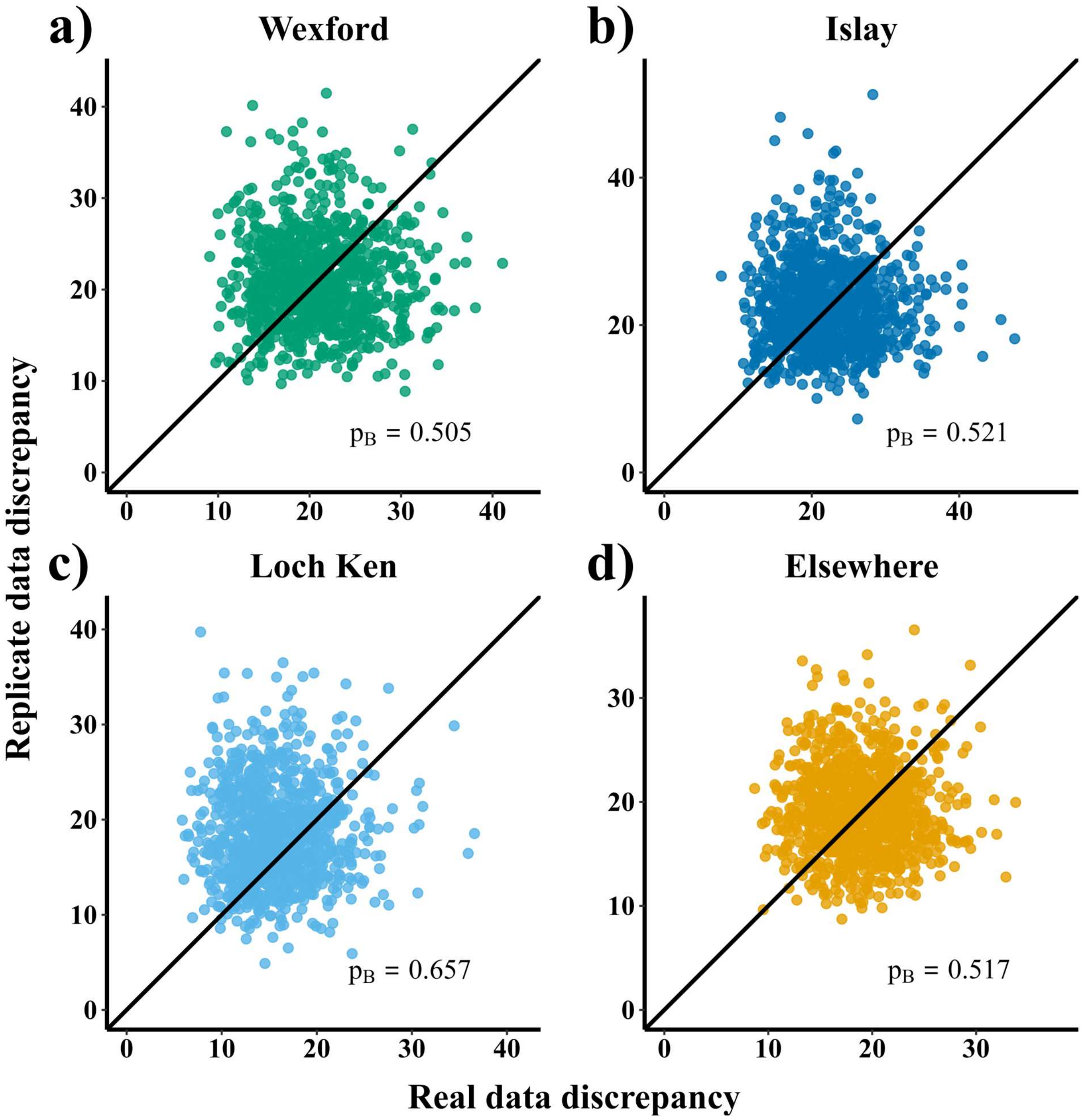

Figure S8. Freeman-Tukey discrepancy measures of expected and observed number of juveniles per subpopulation among all years. Points depict the relationship between the discrepancy measure calculated using real data and the discrepancy measure calculated using each simulated dataset. Bayesian *p*-values (p_B) are defined as the proportion of replicate data sets where the replicate data discrepancy is larger than the real data discrepancy (i.e., proportion of points above the 1:1 line).

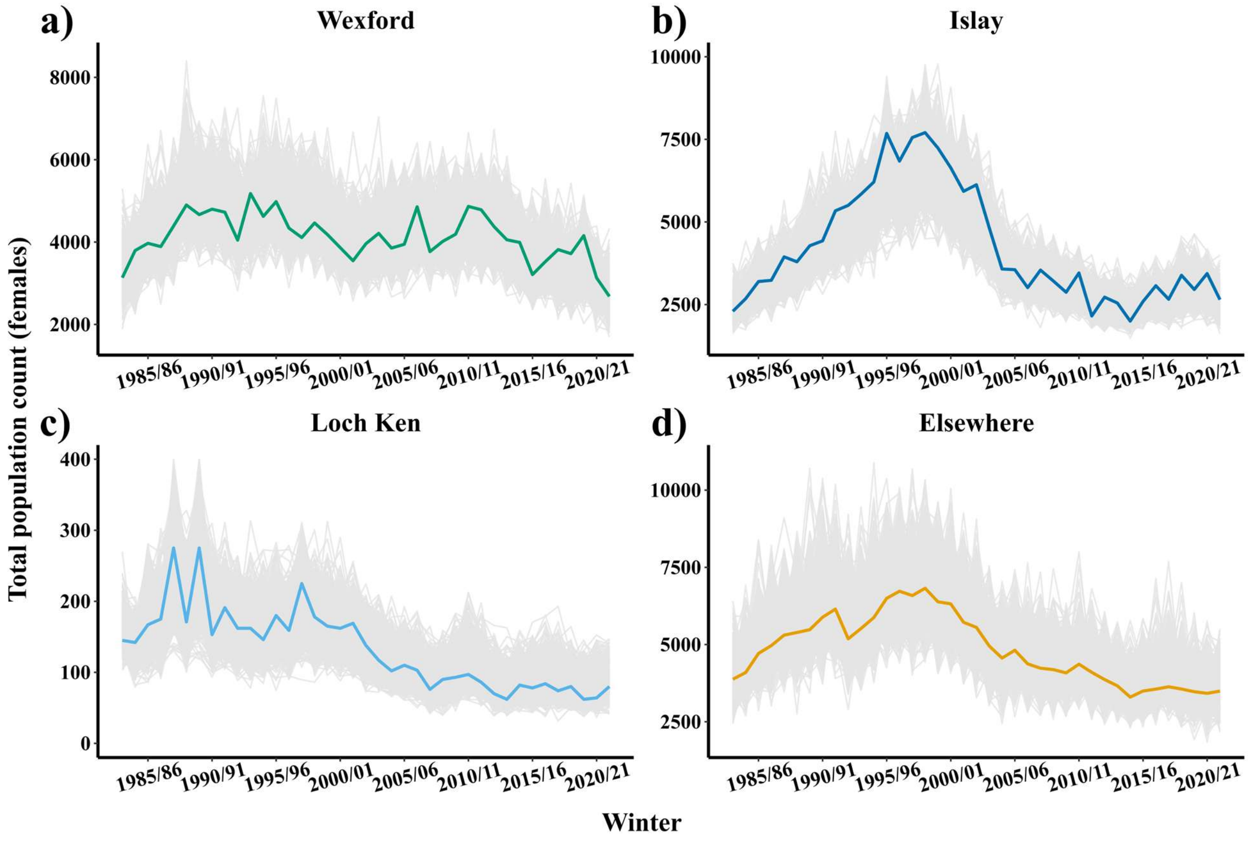

Figure S9. Data used in posterior predictive checks for the population sub-model in the integrated metapopulation model. Coloured lines depict count data and grey lines depict replicate count data generated from 1000 randomly selected posterior draws from estimated demographic rates, movement rates, and observation variation in count data.

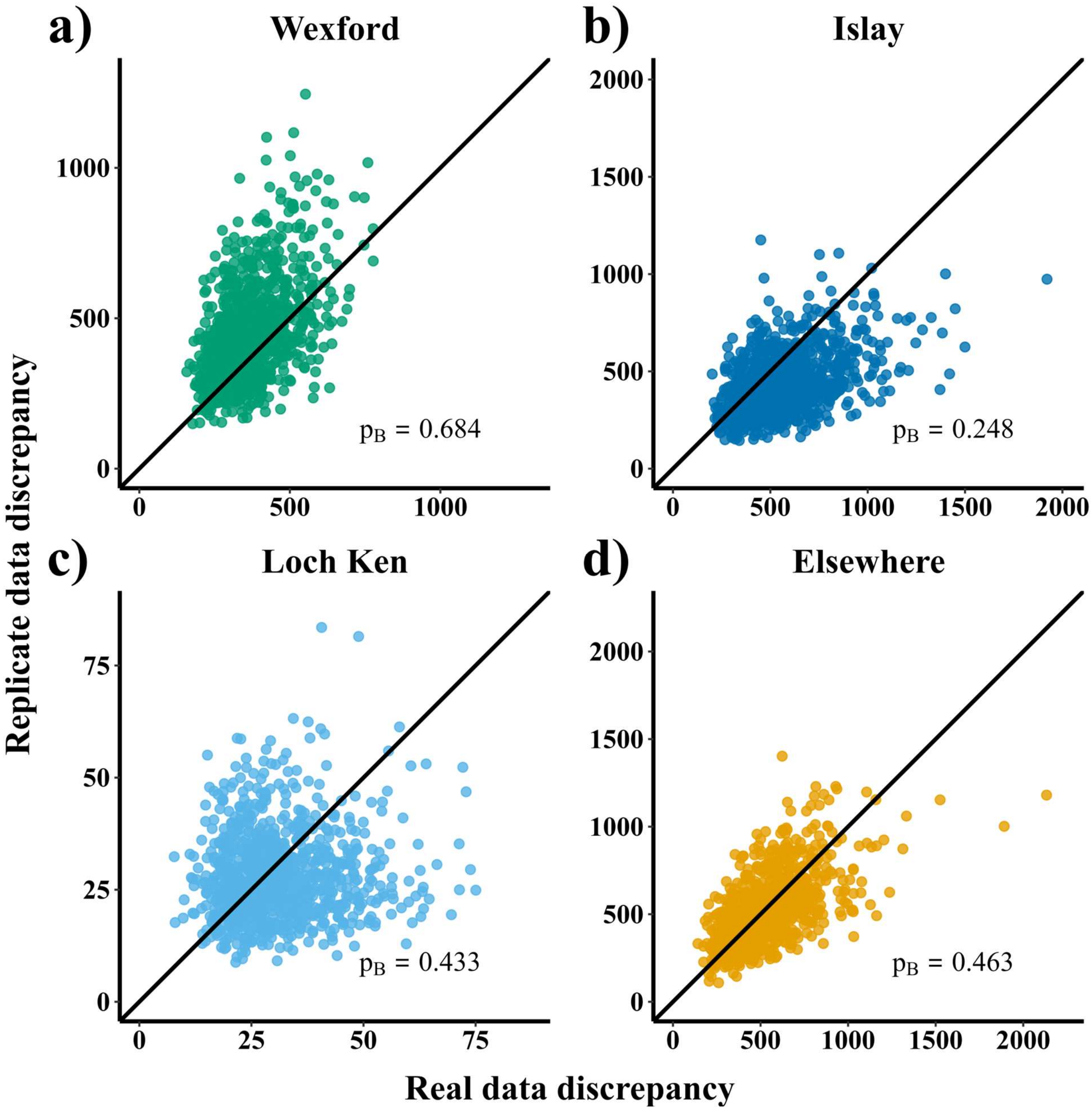

Figure S10. Freeman-Tukey discrepancy measures of expected and observed count per subpopulation among all years. Points depict the relationship between the discrepancy measure calculated using real data and the discrepancy measure calculated using each simulated dataset. Bayesian *p*-values (p_B) are defined as the proportion of replicate data sets where the replicate data discrepancy is larger than the real data discrepancy (i.e., proportion of points above the 1:1 line).

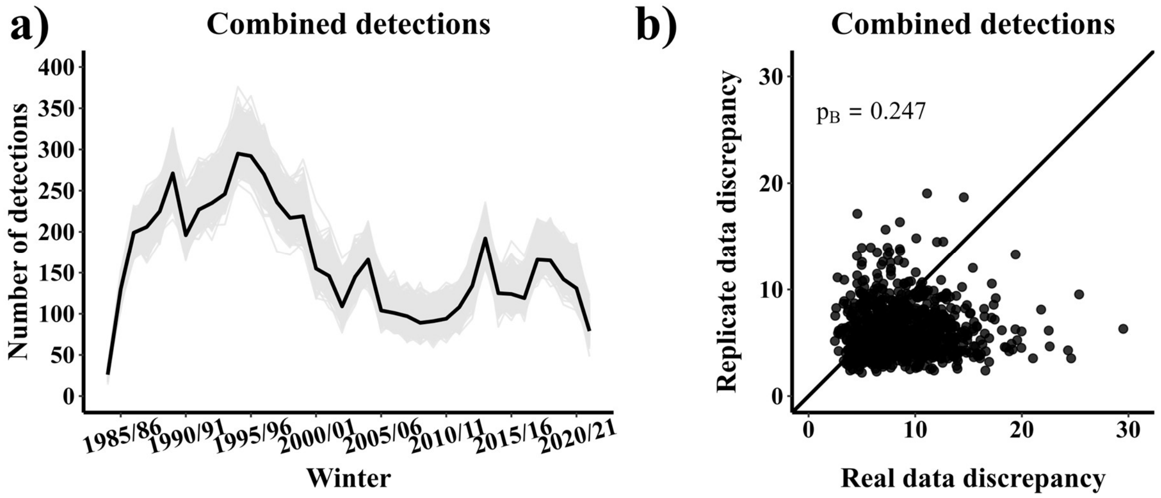

Figure S11. Posterior predictive check for the survival and dispersal sub-model in the integrated metapopulation model. a) Total number of resightings per year from the capture-mark-resighting dataset (black lines) and replicate capture-mark-resighting datasets (grey lines) generated from 1000 randomly selected posterior draws from estimated demographic rates, movement rates, and detection probabilities in the integrated metapopulation model. b) Freeman-Tukey discrepancy measures of expected and observed number of total resightings among all years. Points depict the relationship between the discrepancy measure calculated using real data and the discrepancy measure calculated using each simulated dataset. Bayesian *p*-values (p_B) are defined as the proportion of replicate data sets where the replicate data discrepancy is larger than the real data discrepancy (i.e., proportion of points above the 1:1 line).

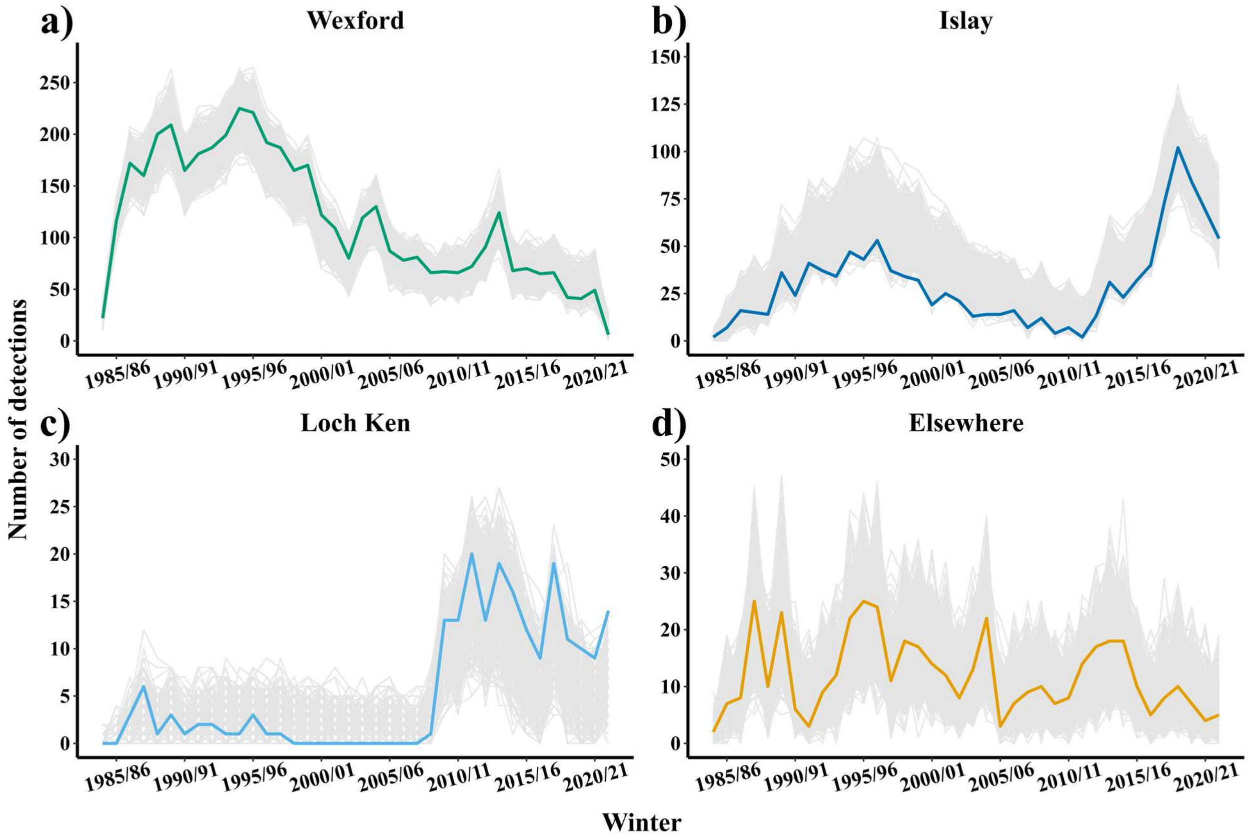

Figure S12. Data used in posterior predictive checks for the survival and dispersal sub-model in the integrated metapopulation model. Coloured lines depict number of resightings per subpopulation and year in the capture-mark-resighting dataset and grey lines depict number of resightings per subpopulation and year in the replicate data generated from 1000 randomly selected posterior draws from estimated demographic rates, movement rates, and detection probabilities.

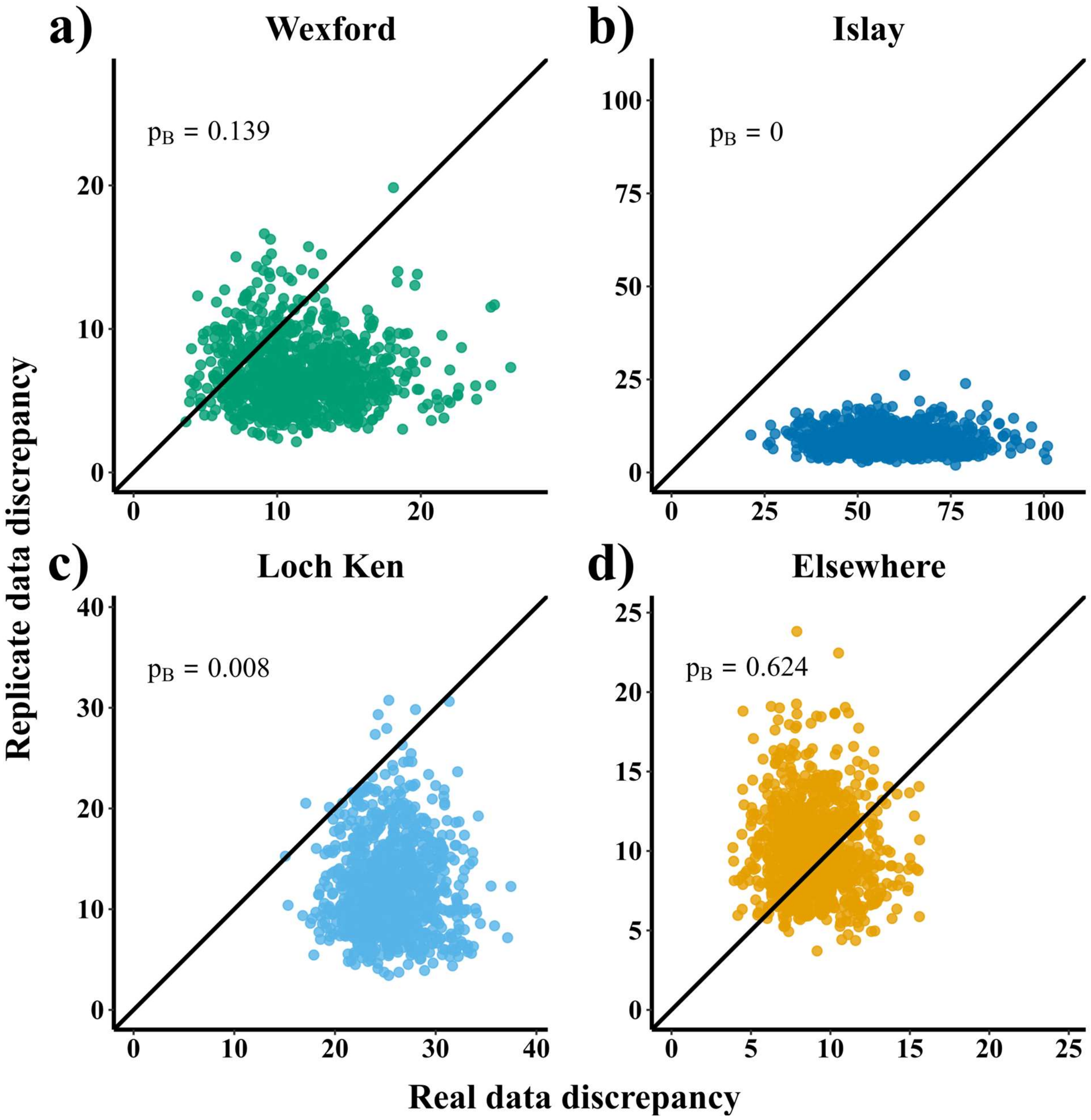

Figures S13. Freeman-Tukey discrepancy measures of expected and observed number of resightings per subpopulation among all years. Points depict the relationship between the discrepancy measure calculated using real data and the discrepancy measure calculated using each simulated dataset. Bayesian *p*-values (p_B) are defined as the proportion of replicate data sets where the replicate data discrepancy is larger than the real data discrepancy (i.e., proportion of points above the 1:1 line).

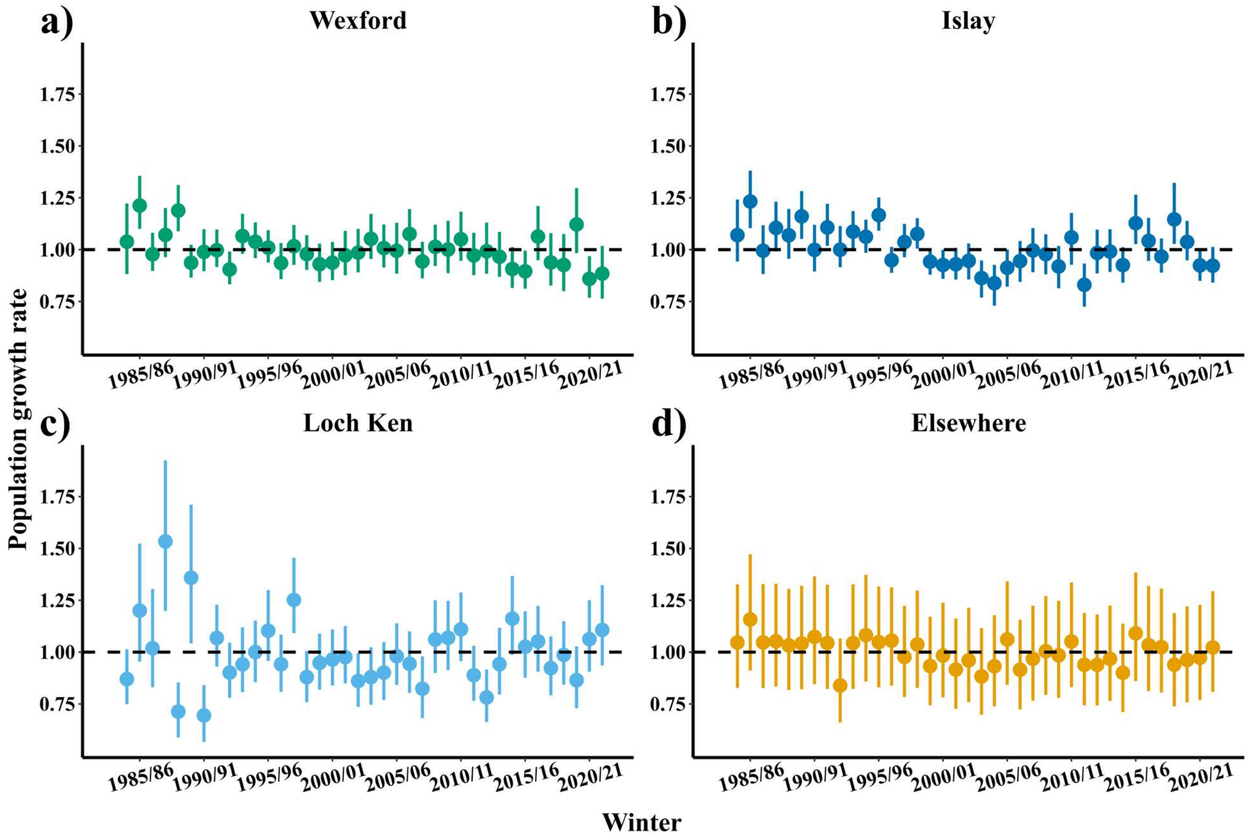

Figure S14. Annual population growth rates (i.e., subpopulation size in year *t* / subpopulation size in year t – 1; posterior medians with 90% credible intervals) for Greenland white-fronted goose females in three focal wintering subpopulations and “Elsewhere.”

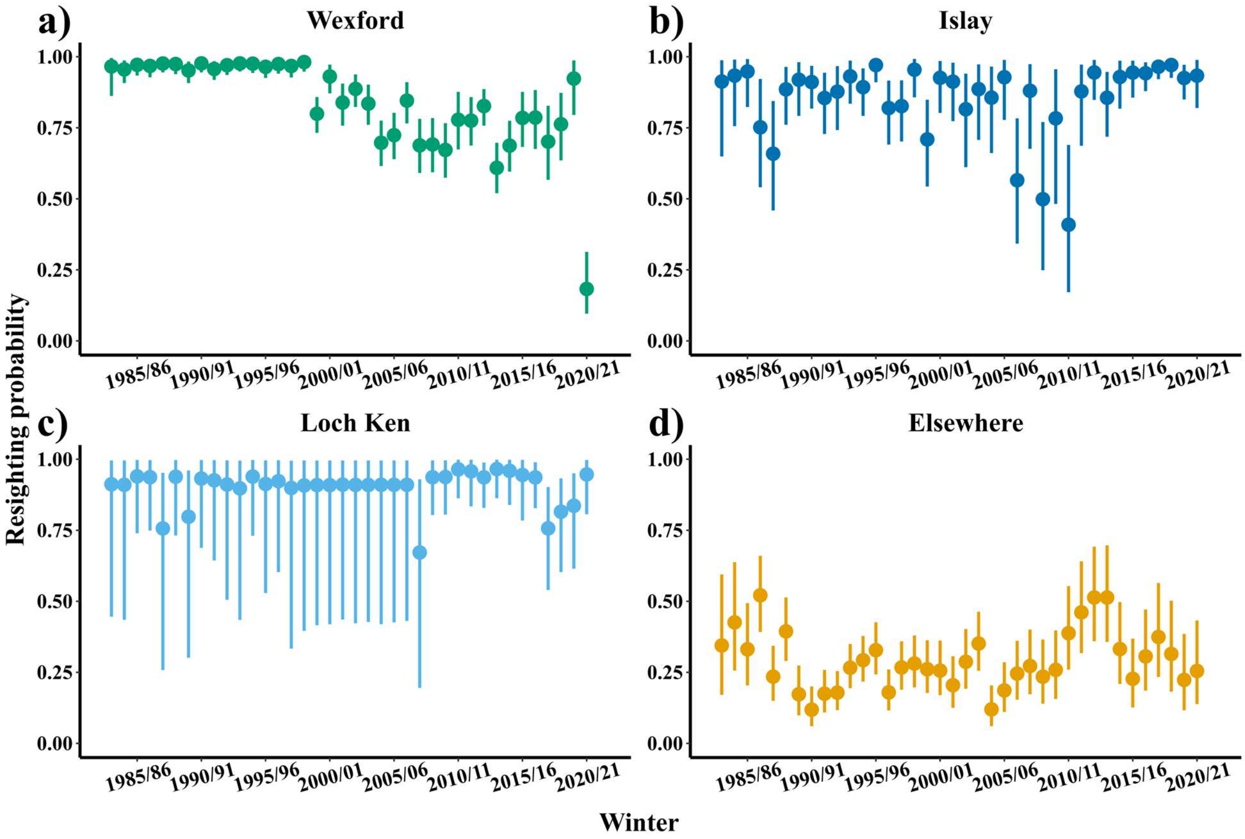

Figure S15. Annual resighting rates (posterior medians with 90% credible intervals) for Greenland white-fronted geese marked with neck collars in three focal subpopulations and Elsewhere.

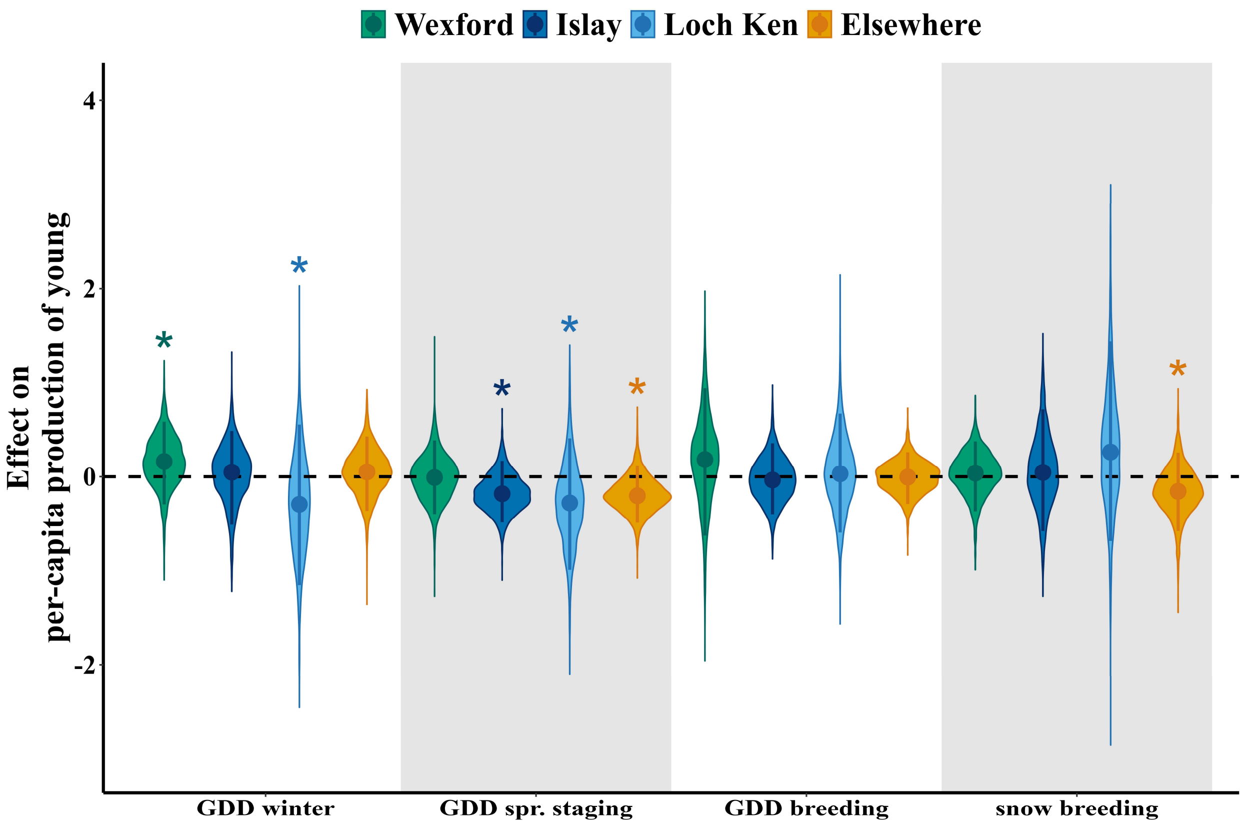

Figure S16. Violin plots depicting the posterior distributions, median estimates (points) and 90% credible intervals (lines) for subpopulation-specific effects of growing degree days on wintering areas (GDD winter), growing degree days on spring staging areas (GDD spr. staging), growing degree days on breeding areas (GDD breeding) and cumulative snow on breeding areas (snow breeding) on per-capita production of young. Y-axis values represent the corresponding β coefficient values on the log scale, interpreted as the log of the ratio of expected per-capita production of young with a one-unit change in the predictor variable (i.e., $\beta = \log(\gamma_{x+1}/\gamma_x)$). Asterisks indicate parameters with >70% of the posterior distribution on the same side of 0 as the mean.

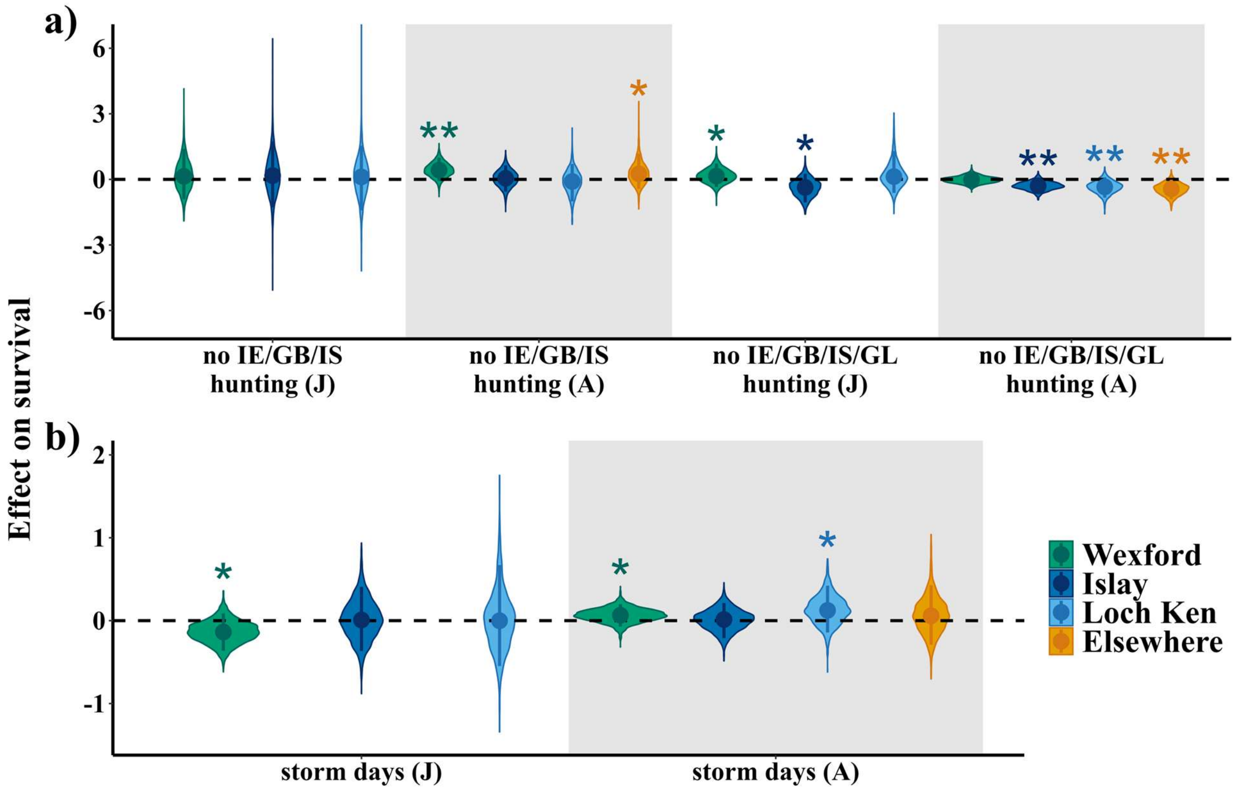

Figure S17. Violin plots depicting the posterior distributions, median estimates (points) and 90% credible intervals (lines) for subpopulation-specific effects of a) hunting protection in Ireland, Great Britain and Iceland (no IE/GB/IS hunting) and hunting protection in Ireland, Great, Britain, Iceland and Greenland (no IE/GB/IS/GL hunting) with respect to hunting protection in Ireland and Great Britain only on juvenile (J) and adult (A) survival and b) number of storm days on juvenile and adult survival. Y-axis values represent the corresponding β coefficient values on the logit scale, interpreted in a) as the log of the survival odds ratio between the corresponding hunting protection category and hunting protection in Ireland and Great Britain only (i.e., $\beta = \log\left(\frac{\varphi_x}{1-\varphi_x} / \frac{\varphi_0}{1-\varphi_0}\right)$) and b) as the log of the survival odds ratio with a one-unit change in storm days (i.e., $\beta = \log\left(\frac{\varphi_{x+1}}{1-\varphi_{x+1}} / \frac{\varphi_x}{1-\varphi_x}\right)$). Single asterisks indicate parameters with >70%, and double asterisks indicate >90% of the posterior distribution on the same side of 0 as the mean.

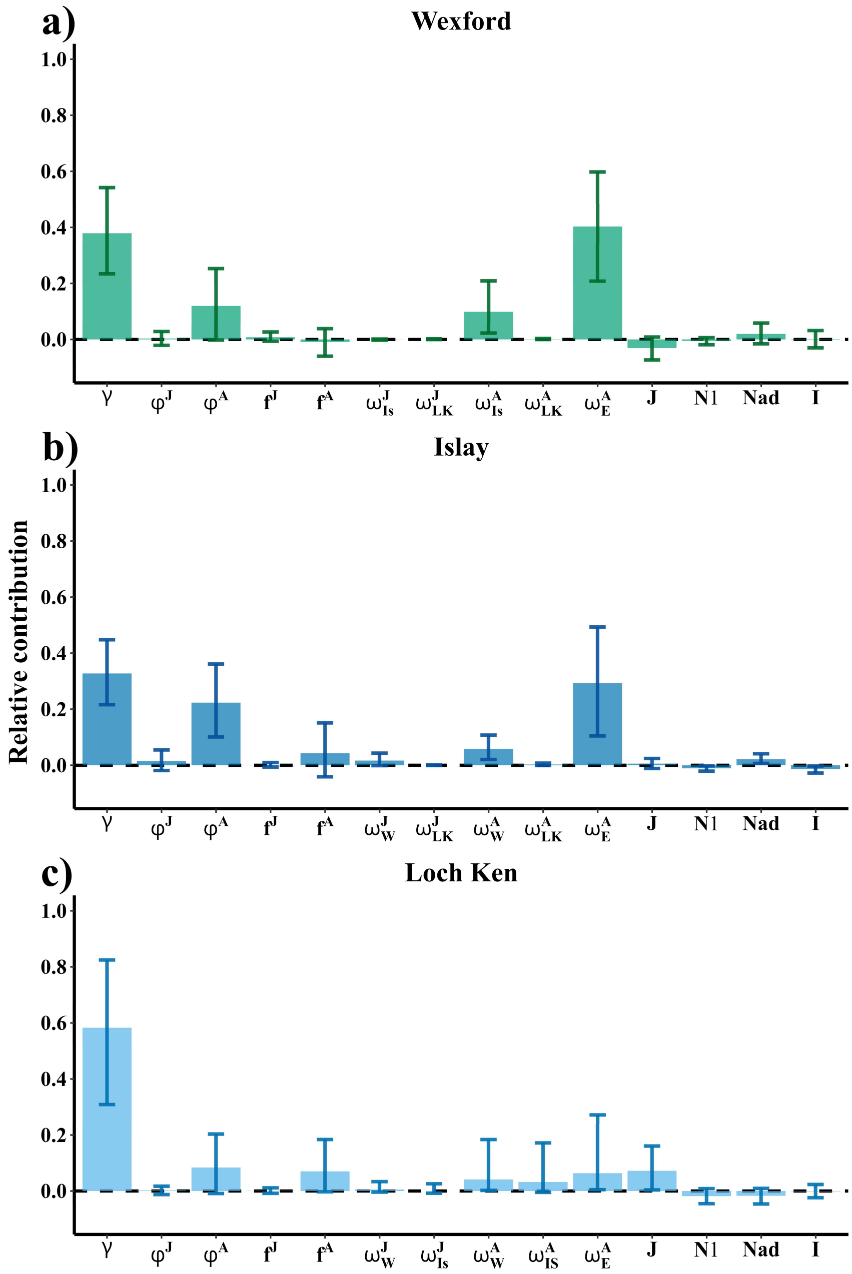

Figure S18. Estimated relative contributions of the realized temporal variation of Greenland white-fronted goose demographic rates (γ = fecundity, φ^J = juvenile survival, φ^A = adult survival, f^J = juvenile fidelity, f^A = adult fidelity), immigration rates (ω_W^J = juvenile immigration rate from Wexford, ω_{Is}^J = juvenile immigration rate from Islay, ω_{LK}^J = juvenile immigration rate from Loch Ken, ω_W^A = adult immigration rate from Wexford, ω_{Is}^A = adult immigration rate from Islay, ω_{LK}^A = adult immigration rate from Loch Ken, ω_E^A = adult immigration rate from Elsewhere), and proportional population structure (J = first winter juveniles, N1 = second winter adults, Nad = third+ winter adults, I = immigrants [all ages]; constrained to sum to 1) to temporal variability of the realised population growth rate for each focal subpopulation. Bars represent posterior medians and error bars represent 90% credible intervals.

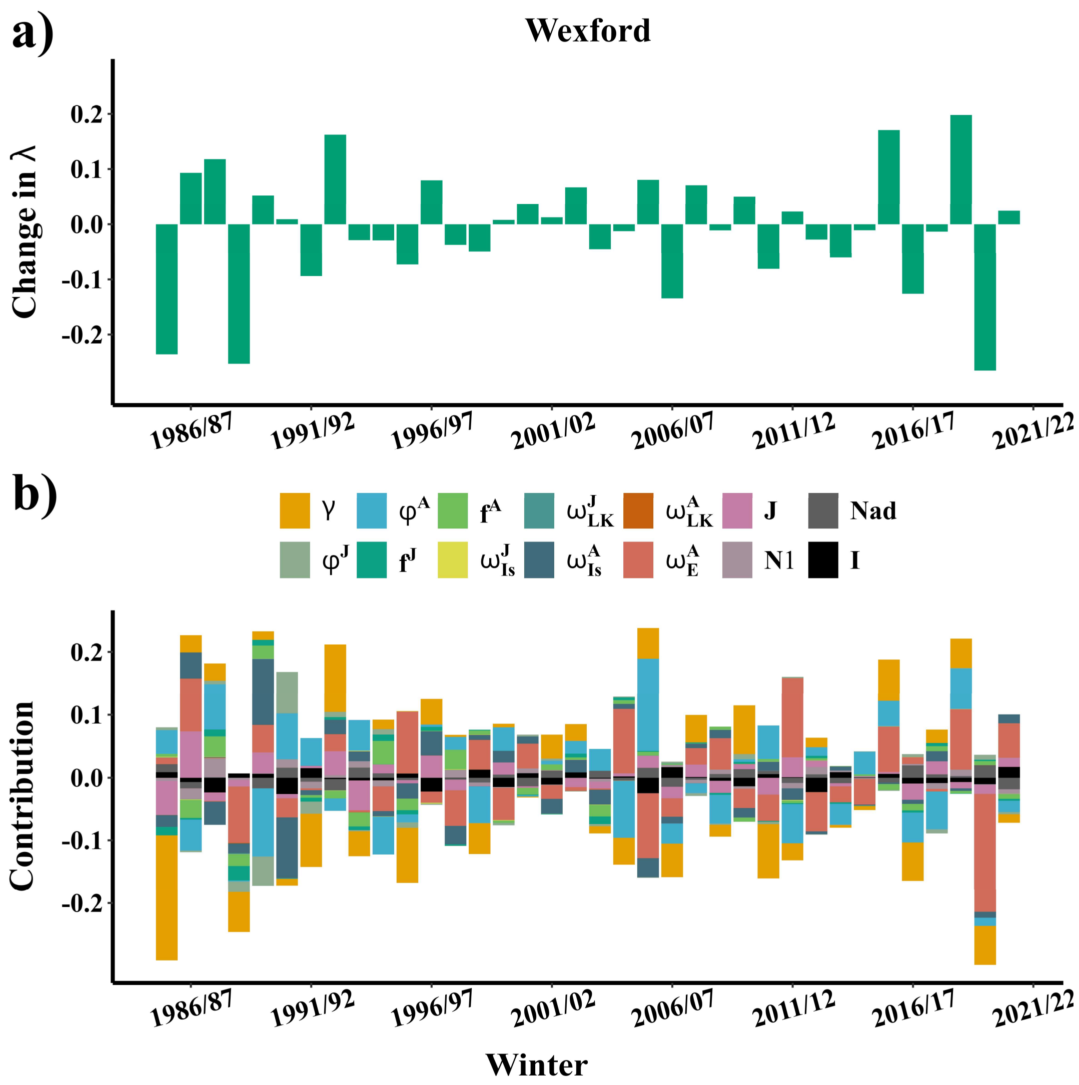

Figure S19. Estimated a) time-sequential changes in realised subpopulation growth rates (λ) for Greenland white-fronted geese wintering in the Wexford subpopulation and b) contributions to these changes from demographic rates (γ = fecundity, φ^J = juvenile survival, φ^A = adult survival, f^J = juvenile fidelity, f^A = adult fidelity), immigration rates (ω_{Is}^J = juvenile immigration rate from Islay, ω_{LK}^J = juvenile immigration rate from Loch Ken, ω_{Is}^A = adult immigration rate from Islay, ω_{LK}^A = adult immigration rate from Loch Ken, ω_E^A = adult immigration rate from Elsewhere), and proportional population structure (J = first winter juveniles, N1 = second winter adults, Nad = third+ winter adults, I = immigrants [all ages]; constrained to sum to 1). Bars represent posterior means. Note that the years on the x-axis represent the first of two successive winters being compared.

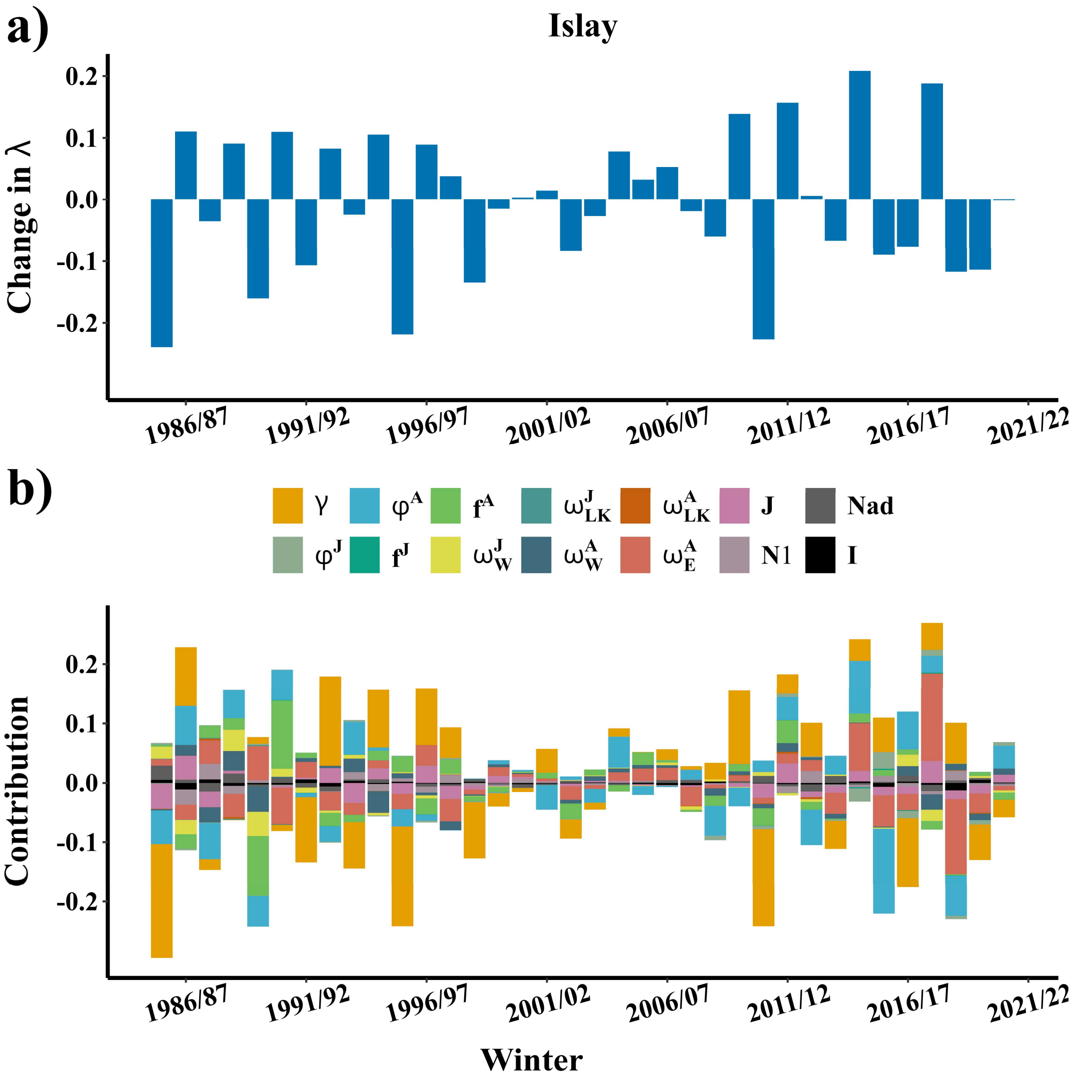

Figure S20. Estimated a) time-sequential changes in realised subpopulation growth rates (λ) for Greenland white-fronted geese wintering in the Islay subpopulation and b) contributions to these changes from demographic rates (γ = fecundity, φ^J = juvenile survival, φ^A = adult survival, f^J = juvenile fidelity, f^A = adult fidelity), immigration rates (ω_W^J = juvenile immigration rate from Wexford, ω_{LK}^J = juvenile immigration rate from Loch Ken, ω_W^A = adult immigration rate from Wexford, ω_{LK}^A = adult immigration rate from Loch Ken, ω_E^A = adult immigration rate from Elsewhere), and proportional population structure (J = first winter juveniles, N1 = second winter adults, Nad = third+ winter adults, I = immigrants [all ages]; constrained to sum to 1). Bars represent posterior means. Note that the years on the x-axis represent the first of two successive winters being compared.

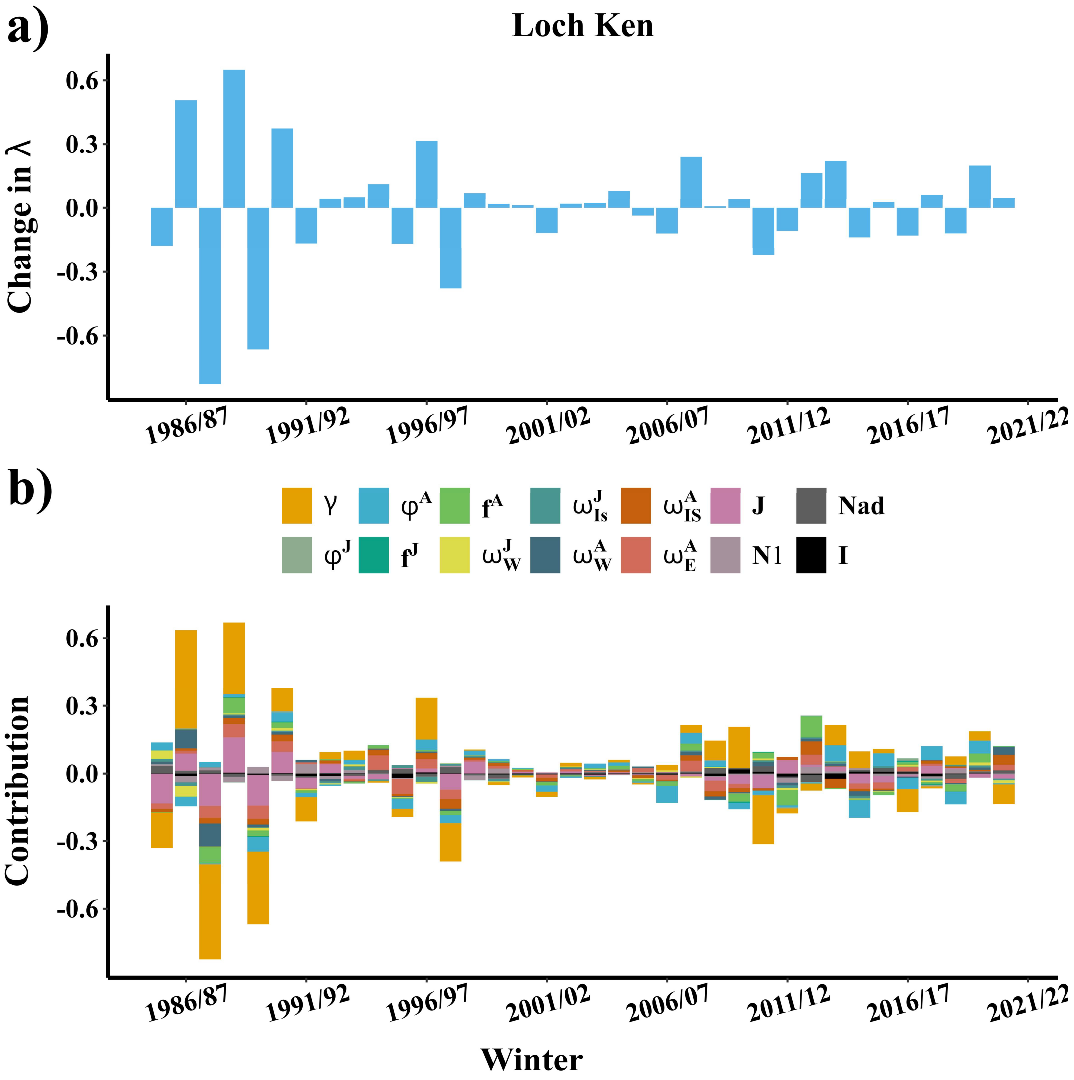

Figure S21. Estimated a) time-sequential changes in realised subpopulation growth rates (λ) for Greenland white-fronted geese wintering in the Loch Ken subpopulation and b) contributions to these changes from demographic rates (γ = fecundity, φ^J = juvenile survival, φ^A = adult survival, f^J = juvenile fidelity, f^A = adult fidelity), immigration rates (ω_W^J = juvenile immigration rate from Wexford, ω_{IS}^J = juvenile immigration rate from Islay, ω_W^A = adult immigration rate from Wexford, ω_{IS}^A = adult immigration rate from Islay, ω_E^A = adult immigration rate from Elsewhere), and proportional population structure (J = first winter juveniles, N1 = second winter adults, Nad = third+ winter adults, I = immigrants [all ages]; constrained to sum to 1). Bars represent posterior means. Note that the years on the x-axis represent the first of two successive winters being compared.

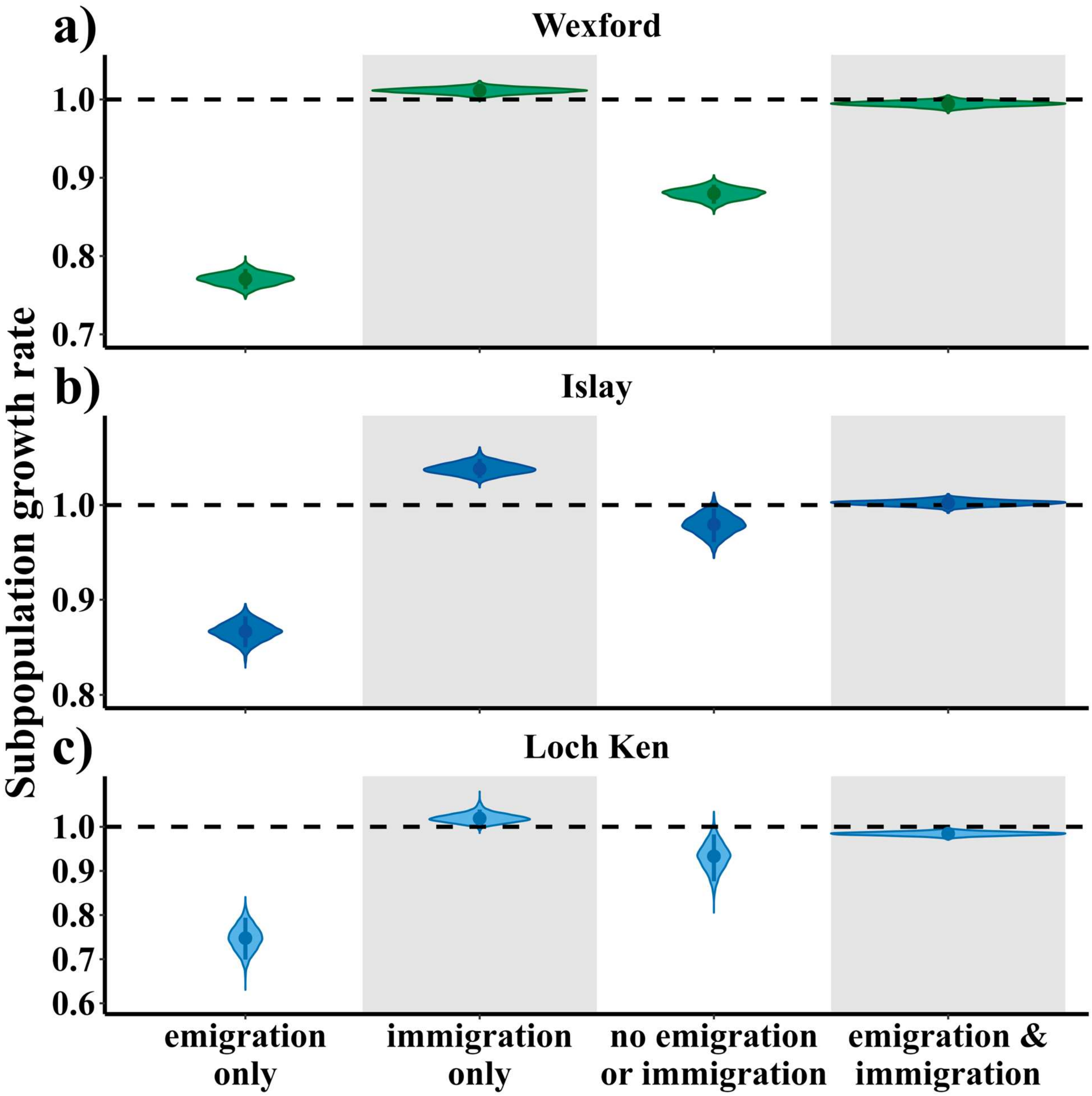

Figure S22. Violin plots depicting projected population growth rates of each focal subpopulation with emigration only, no emigration or immigration, emigration and immigration and emigration only. Points are the median estimates and vertical lines are 90% credible intervals.
